# Supplementary material for: Connectivity of the right cerebello-left hippocampal circuit across adulthood
Source: Imaging Neurosci (Camb). 2026 May 15;4:IMAG.a.1233. doi: 10.1162/IMAG.a.1233 (PMC13181821; doi:10.1162/IMAG.a.1233)
Supplement: Supplementary Material [file IMAG.a.1233_supp.pdf]

Supplement

Supplementary Table 1. Demographic means for the full sample are listed below with standard deviations in parentheses.

**Demographics**	
Age (years)	57(13.31)
Sex F(M)	74(64)
Education (years)	16.7 (2.3)
African American (%)	2%
Asian (%)	7%
Caucasian (%)	86%
Multiracial (%)	4%
Native American (%)	1%

Supplementary Table 2. Cerebellar and Hippocampal Coordinates for 5mm Spherical Seeds

**Seed Label**	**X**	**Y**	**Z**	**Region (AAL)**
R_Medial boundary_Xaxis	5.00	-59.20	-36.50	Vermis IX
R_Lateral_boundary_Xaxis	51.30	-59.10	-36.50	Crus I
R_Caudal_boundary_Yaxis	0.00	-74.70	-26.00	Vermis VII
R_Rostral_boundary_Yaxis	0.20	-50.30	-26.00	Vermis X
R_Dorsal_boundary_Zaxis	0.80	-55.40	-3.90	Vermis IV-V
R_Ventral_boundary_Zaxis	1.00	-55.50	-51.10	Lobule IX
R_CB1	15.00	-74.60	-51.10	Lobule VIII
R_CB2	25.00	-74.60	-51.10	Lobule VIIb
R_CB3	35.00	-74.60	-51.10	Lobule VIIb
R_CB4	15.00	-64.60	-51.10	Lobule VIII
R_CB5	25.00	-64.60	-51.10	Lobule VIII
R_CB6	35.00	-64.60	-51.10	Lobule VIII
R_CB7	15.00	-54.60	-51.10	Lobule IX
R_CB8	25.00	-54.60	-51.10	Lobule VIII
R_CB9	35.00	-54.60	-51.10	Lobule VIII
R_CB10	45.00	-54.60	-51.10	Lobule VIIb
R_CB11	15.00	-74.60	-41.10	Lobule VIIb
R_CB12	25.00	-74.60	-41.10	Crus II
R_CB13	35.00	-74.60	-41.10	Crus II
R_CB14	15.00	-64.60	-41.10	Lobule VIII
R_CB15	25.00	-64.60	-41.10	Lobule VIII
R_CB16	35.00	-64.60	-41.10	Crus II
R_CB17	45.00	-64.60	-41.10	Crus II
R_CB18	15.00	-54.60	-41.10	Lobule VIII
R_CB19	25.00	-54.60	-41.10	Lobule VIII
R_CB20	35.00	-54.60	-41.10	Crus I
R_CB21	45.00	-54.60	-41.10	Crus II

R_CB22	15.00	-74.60	-31.10	Crus I
R_CB23	25.00	-74.60	-31.10	Crus I
R_CB24	35.00	-74.60	-31.10	Crus I
R_CB25	45.00	-74.60	-31.10	Crus I
R_CB26	15.00	-64.60	-31.10	Lobule VI
R_CB27	25.00	-64.60	-31.10	Lobule VI
R_CB28	35.00	-64.60	-31.10	Crus I
R_CB29	45.00	-64.60	-31.10	Crus I
R_CB30	15.00	-54.60	-31.10	Lobule VI
R_CB31	25.00	-54.60	-31.10	Lobule VI
R_CB32	35.00	-54.60	-31.10	Crus I
R_CB33	45.00	-54.60	-31.10	Crus I
R_CB34	15.00	-74.60	-21.10	Lobule VI
R_CB35	5.00	-64.60	-21.10	Vermis VI
R_CB36	15.00	-64.60	-21.10	Lobule VI
R_CB37	5.00	-54.60	-21.10	Vermis IV-V
R_CB38	15.00	-54.60	-21.10	Lobule VI
R_CB39	5.00	-64.60	-11.10	Vermis VI
Hipp_L_Ant	-30.00	-10.00	-22.00	Hippocampus
Hipp_L_Ant_1	-24.00	-18.00	-16.00	Hippocampus
Hipp_L_Ant_2	-33.00	-18.00	-18.00	Hippocampus
Hipp_L_Ant_3_Rev2	-19.00	-10.00	-22.00	Parahippocampal gyrus
Hipp_L_Post1	-32.00	-36.00	-5.00	Hippocampus
Hipp_L_Post2	-33.00	-27.00	-11.00	Hippocampus
Hipp_L_Post3	-27.00	-32.00	-11.00	Parahippocampal gyrus

Supplementary Figure 1. Cerebellar Seeds Rendered in marsbar.

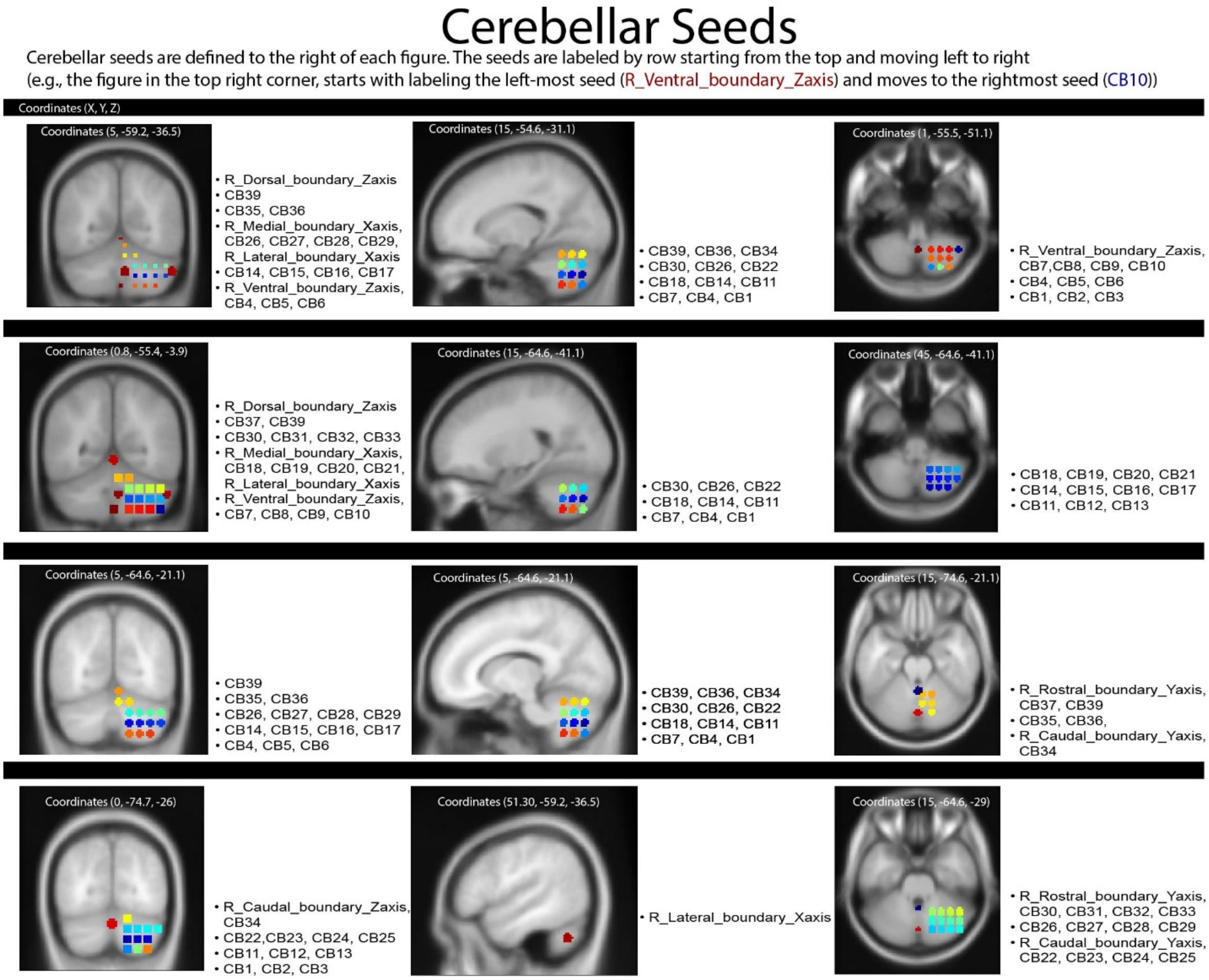

Supplementary Table 3. Cerebellar-hippocampal regions showing significant correlations when controlling for age.

FC Seed Regions		T(136)	pFDR	Cohen's d
R_Dorsal_boundary_Zaxis	Hipp_L_Ant_2	-4.07	0.002382	-0.70
Hipp_L_Ant_2	R_Dorsal_boundary_Zaxis	-4.07	0.002382	-0.70
Hipp_L_Ant_2	R_CB36	-3.84	0.002382	-0.66
R_Dorsal_boundary_Zaxis	Hipp_L_Ant	-3.82	0.002382	-0.66
R_CB36	Hipp_L_Ant_2	-3.84	0.002382	-0.66
Hipp_L_Ant	R_Dorsal_boundary_Zaxis	-3.82	0.002382	-0.66
Hipp_L_Ant	R_CB38	-3.49	0.005873	-0.60
Hipp_L_Ant	R_CB36	-3.33	0.005873	-0.57
R_CB38	Hipp_L_Ant	-3.49	0.00805	-0.60
R_CB36	Hipp_L_Ant	-3.33	0.00805	-0.57

Hipp_L_Ant_2	R_CB30	-3.07	0.013939	-0.53
R_Dorsal_boundary_Zaxis	Hipp_L_Ant_1	-3.05	0.013939	-0.52
Hipp_L_Ant_2	R_CB38	-2.81	0.013939	-0.48
Hipp_L_Ant_2	R_CB18	-2.77	0.013939	-0.48
Hipp_L_Ant	R_CB4	-2.64	0.014398	-0.45
Hipp_L_Ant	R_CB7	-2.62	0.014398	-0.45
Hipp_L_Ant_2	R_CB26	-2.67	0.016437	-0.46
Hipp_L_Ant_2	R_CB8	-2.62	0.016437	-0.45
R_CB36	Hipp_L_Ant_1	-2.88	0.016437	-0.49
R_CB36	Hipp_L_Ant_3_Rev2	-2.76	0.016437	-0.47
R_CB36	Hipp_L_Post3	-2.75	0.016437	-0.47
Hipp_L_Ant_2	R_CB31	-2.47	0.016437	-0.42
Hipp_L_Post3	R_Caudal_boundary_Yaxis	-3.00	0.016437	-0.51
Hipp_L_Post3	R_CB35	-2.87	0.016437	-0.49
R_Caudal_boundary_Yaxis	Hipp_L_Post3	-3.00	0.016437	-0.51
Hipp_L_Post3	R_CB36	-2.75	0.016437	-0.47
R_CB38	Hipp_L_Ant_2	-2.81	0.016437	-0.48
Hipp_L_Ant_2	R_CB15	-2.22	0.016437	-0.38
R_CB35	Hipp_L_Post3	-2.87	0.016437	-0.49
R_CB35	Hipp_L_Ant_3_Rev2	-2.81	0.016437	-0.48
R_CB35	Hipp_L_Ant_1	-2.71	0.016877	-0.46
Hipp_L_Ant_1	R_Dorsal_boundary_Zaxis	-3.05	0.016877	-0.52
Hipp_L_Ant_1	R_CB36	-2.88	0.017632	-0.49
Hipp_L_Ant	R_Medial_boundary_Xaxis	-2.24	0.017632	-0.38
Hipp_L_Ant_1	R_CB35	-2.71	0.017632	-0.46
R_CB30	Hipp_L_Ant_2	-3.07	0.017632	-0.53
R_Dorsal_boundary_Zaxis	Hipp_L_Ant_3_Rev2	-2.34	0.017632	-0.40
R_Dorsal_boundary_Zaxis	Hipp_L_Post1	2.25	0.017632	0.39
Hipp_L_Ant	R_CB18	-2.04	0.017632	-0.35
Hipp_L_Post3	R_CB38	-2.48	0.017632	-0.43
R_CB18	Hipp_L_Ant_2	-2.77	0.024152	-0.48
Hipp_L_Ant_3_Rev2	R_CB35	-2.81	0.024152	-0.48
Hipp_L_Ant_3_Rev2	R_CB36	-2.76	0.024152	-0.47
R_CB38	Hipp_L_Post3	-2.48	0.024152	-0.43
Hipp_L_Ant_3_Rev2	R_Dorsal_boundary_Zaxis	-2.34	0.031141	-0.40
R_Caudal_boundary_Yaxis	Hipp_L_Ant_1	-2.34	0.031141	-0.40
R_Caudal_boundary_Yaxis	Hipp_L_Ant_3_Rev2	-2.25	0.031141	-0.39
Hipp_L_Ant_3_Rev2	R_Caudal_boundary_Yaxis	-2.25	0.031141	-0.39
Hipp_L_Ant_1	R_Caudal_boundary_Yaxis	-2.34	0.034154	-0.40
Hipp_L_Ant_1	R_CB31	-2.28	0.034154	-0.39
Hipp_L_Ant_1	R_CB30	-2.16	0.034154	-0.37
R_CB31	Hipp_L_Ant_2	-2.47	0.034154	-0.42
R_CB31	Hipp_L_Ant_1	-2.28	0.034154	-0.39
Hipp_L_Post3	R_CB8	-2.17	0.034154	-0.37
Hipp_L_Post3	R_CB7	-2.12	0.034154	-0.36

Hipp_L_Ant_1	R_CB38	-2.02	0.034154	-0.35
R_CB4	Hipp_L_Ant	-2.64	0.034782	-0.45
R_CB8	Hipp_L_Ant_2	-2.62	0.034782	-0.45
R_CB8	Hipp_L_Post3	-2.17	0.038173	-0.37
R_CB8	Hipp_L_Post2	-2.09	0.038173	-0.36
R_CB7	Hipp_L_Ant	-2.62	0.038173	-0.45
R_CB26	Hipp_L_Ant_2	-2.67	0.038173	-0.46
R_CB7	Hipp_L_Post3	-2.12	0.039912	-0.36
R_CB18	Hipp_L_Ant	-2.04	0.039912	-0.35
R_CB38	Hipp_L_Ant_1	-2.02	0.042022	-0.35
R_CB38	Hipp_L_Post2	-1.99	0.042022	-0.34
R_CB30	Hipp_L_Ant_1	-2.16	0.045879	-0.37
R_CB15	Hipp_L_Ant_2	-2.22	0.045879	-0.38
R_Medial_boundary_Xaxis	Hipp_L_Ant	-2.24	0.046119	-0.38
Hipp_L_Post2	R_CB8	-2.09	0.046119	-0.36
Hipp_L_Post2	R_CB38	-1.99	0.048173	-0.34
Hipp_L_Post1	R_Dorsal_boundary_Zaxis	2.25	0.048173	0.39
R_Dorsal_boundary_Zaxis	Hipp_L_Ant_2	-4.07	0.002382	-0.70
Hipp_L_Ant_2	R_Dorsal_boundary_Zaxis	-4.07	0.002382	-0.70
Hipp_L_Ant_2	R_CB36	-3.84	0.002382	-0.66
R_Dorsal_boundary_Zaxis	Hipp_L_Ant	-3.82	0.002382	-0.66
R_CB36	Hipp_L_Ant_2	-3.84	0.002382	-0.66
Hipp_L_Ant	R_Dorsal_boundary_Zaxis	-3.82	0.002382	-0.66
Hipp_L_Ant	R_CB38	-3.49	0.005873	-0.60
Hipp_L_Ant	R_CB36	-3.33	0.005873	-0.57
R_CB38	Hipp_L_Ant	-3.49	0.00805	-0.60
R_CB36	Hipp_L_Ant	-3.33	0.00805	-0.57
Hipp_L_Ant_2	R_CB30	-3.07	0.013939	-0.53
R_Dorsal_boundary_Zaxis	Hipp_L_Ant_1	-3.05	0.013939	-0.52
Hipp_L_Ant_2	R_CB38	-2.81	0.013939	-0.48
Hipp_L_Ant_2	R_CB18	-2.77	0.013939	-0.48
Hipp_L_Ant	R_CB4	-2.64	0.014398	-0.45
Hipp_L_Ant	R_CB7	-2.62	0.014398	-0.45
Hipp_L_Ant_2	R_CB26	-2.67	0.016437	-0.46
Hipp_L_Ant_2	R_CB8	-2.62	0.016437	-0.45
R_CB36	Hipp_L_Ant_1	-2.88	0.016437	-0.49
R_CB36	Hipp_L_Ant_3_Rev2	-2.76	0.016437	-0.47
R_CB36	Hipp_L_Post3	-2.75	0.016437	-0.47
Hipp_L_Ant_2	R_CB31	-2.47	0.016437	-0.42
Hipp_L_Post3	R_Caudal_boundary_Yaxis	-3.00	0.016437	-0.51
Hipp_L_Post3	R_CB35	-2.87	0.016437	-0.49
R_Caudal_boundary_Yaxis	Hipp_L_Post3	-3.00	0.016437	-0.51
Hipp_L_Post3	R_CB36	-2.75	0.016437	-0.47
R_CB38	Hipp_L_Ant_2	-2.81	0.016437	-0.48
Hipp_L_Ant_2	R_CB15	-2.22	0.016437	-0.38

R_CB35	Hipp_L_Post3	-2.87	0.016437	-0.49
R_CB35	Hipp_L_Ant_3_Rev2	-2.81	0.016437	-0.48
R_CB35	Hipp_L_Ant_1	-2.71	0.016877	-0.46
Hipp_L_Ant_1	R_Dorsal_boundary_Zaxis	-3.05	0.016877	-0.52
Hipp_L_Ant_1	R_CB36	-2.88	0.017632	-0.49
Hipp_L_Ant	R_Medial_boundary_Xaxis	-2.24	0.017632	-0.38
Hipp_L_Ant_1	R_CB35	-2.71	0.017632	-0.46
R_CB30	Hipp_L_Ant_2	-3.07	0.017632	-0.53
R_Dorsal_boundary_Zaxis	Hipp_L_Ant_3_Rev2	-2.34	0.017632	-0.40
R_Dorsal_boundary_Zaxis	Hipp_L_Post1	2.25	0.017632	0.39
Hipp_L_Ant	R_CB18	-2.04	0.017632	-0.35
Hipp_L_Post3	R_CB38	-2.48	0.017632	-0.43
R_CB18	Hipp_L_Ant_2	-2.77	0.024152	-0.48
Hipp_L_Ant_3_Rev2	R_CB35	-2.81	0.024152	-0.48
Hipp_L_Ant_3_Rev2	R_CB36	-2.76	0.024152	-0.47
R_CB38	Hipp_L_Post3	-2.48	0.024152	-0.43
Hipp_L_Ant_3_Rev2	R_Dorsal_boundary_Zaxis	-2.34	0.031141	-0.40
R_Caudal_boundary_Yaxis	Hipp_L_Ant_1	-2.34	0.031141	-0.40
R_Caudal_boundary_Yaxis	Hipp_L_Ant_3_Rev2	-2.25	0.031141	-0.39
Hipp_L_Ant_3_Rev2	R_Caudal_boundary_Yaxis	-2.25	0.031141	-0.39
Hipp_L_Ant_1	R_Caudal_boundary_Yaxis	-2.34	0.034154	-0.40
Hipp_L_Ant_1	R_CB31	-2.28	0.034154	-0.39
Hipp_L_Ant_1	R_CB30	-2.16	0.034154	-0.37
R_CB31	Hipp_L_Ant_2	-2.47	0.034154	-0.42
R_CB31	Hipp_L_Ant_1	-2.28	0.034154	-0.39
Hipp_L_Post3	R_CB8	-2.17	0.034154	-0.37
Hipp_L_Post3	R_CB7	-2.12	0.034154	-0.36
Hipp_L_Ant_1	R_CB38	-2.02	0.034154	-0.35
R_CB4	Hipp_L_Ant	-2.64	0.034782	-0.45
R_CB8	Hipp_L_Ant_2	-2.62	0.034782	-0.45
R_CB8	Hipp_L_Post3	-2.17	0.038173	-0.37
R_CB8	Hipp_L_Post2	-2.09	0.038173	-0.36
R_CB7	Hipp_L_Ant	-2.62	0.038173	-0.45
R_CB26	Hipp_L_Ant_2	-2.67	0.038173	-0.46
R_CB7	Hipp_L_Post3	-2.12	0.039912	-0.36
R_CB18	Hipp_L_Ant	-2.04	0.039912	-0.35
R_CB38	Hipp_L_Ant_1	-2.02	0.042022	-0.35
R_CB38	Hipp_L_Post2	-1.99	0.042022	-0.34
R_CB30	Hipp_L_Ant_1	-2.16	0.045879	-0.37
R_CB15	Hipp_L_Ant_2	-2.22	0.045879	-0.38
R_Medial_boundary_Xaxis	Hipp_L_Ant	-2.24	0.046119	-0.38
Hipp_L_Post2	R_CB8	-2.09	0.046119	-0.36
Hipp_L_Post2	R_CB38	-1.99	0.048173	-0.34
Hipp_L_Post1	R_Dorsal_boundary_Zaxis	2.25	0.048173	0.39

Supplementary Table 4. Cerebellar-hippocampal regions showing significant correlations with increased 17β-estradiol levels.

FC Seed Regions		T(126)	pFDR	Cohen's d
R_CB36	Hipp_L_Ant_2	3.51	0.0147	0.63
Hipp_L_Ant_2	R_CB36	3.51	0.0147	0.63
R_CB35	Hipp_L_Ant_3_Rev2	3.25	0.0147	0.58
Hipp_L_Ant_2	R_Dorsal_boundary_Zaxis	3.15	0.0147	0.56
Hipp_L_Ant_2	R_CB30	3.07	0.0147	0.55
R_CB36	Hipp_L_Ant_1	3.12	0.0147	0.56
Hipp_L_Ant_2	R_CB8	2.74	0.0147	0.49
Hipp_L_Ant_3_Rev2	R_CB35	3.25	0.0147	0.58
R_CB35	Hipp_L_Ant_1	2.88	0.0147	0.51
R_Dorsal_boundary_Zaxis	Hipp_L_Ant_2	3.15	0.0147	0.56
Hipp_L_Ant_1	R_CB36	3.12	0.0217	0.56
Hipp_L_Ant_1	R_CB35	2.88	0.0217	0.51
R_CB30	Hipp_L_Ant_2	3.07	0.0231	0.55
R_CB38	Hipp_L_Ant	2.81	0.0231	0.50
Hipp_L_Ant_1	R_Medial_boundary_Xaxis	2.65	0.0243	0.47
R_CB38	Hipp_L_Post3	2.62	0.0243	0.47
R_CB38	Hipp_L_Ant_1	2.58	0.0253	0.46
Hipp_L_Ant_1	R_CB38	2.58	0.0253	0.46
Hipp_L_Ant_2	R_CB31	2.38	0.0253	0.42
R_CB8	Hipp_L_Ant_2	2.74	0.0253	0.49
Hipp_L_Ant	R_CB38	2.81	0.0253	0.50
Hipp_L_Ant	R_CB36	2.34	0.0253	0.42
Hipp_L_Ant	R_CB18	2.23	0.0258	0.40
Hipp_L_Ant	R_CB7	2.22	0.0258	0.40
Hipp_L_Ant	R_Dorsal_boundary_Zaxis	2.21	0.0285	0.39
Hipp_L_Ant	R_Medial_boundary_Xaxis	2.14	0.0285	0.38
R_CB36	Hipp_L_Post3	2.35	0.0363	0.42
R_CB36	Hipp_L_Ant	2.34	0.0363	0.42
R_CB37	Hipp_L_Post1	-2.65	0.0363	-0.47
Hipp_L_Ant_2	R_CB15	2.03	0.0363	0.36
R_CB35	Hipp_L_Post3	2.18	0.0363	0.39
Hipp_L_Ant_1	R_Dorsal_boundary_Zaxis	2.07	0.0363	0.37
Hipp_L_Ant_1	R_CB14	2.06	0.0431	0.37
Hipp_L_Ant_1	R_CB31	2.06	0.0431	0.37
Hipp_L_Ant_1	R_CB30	2.06	0.0431	0.37
R_Dorsal_boundary_Zaxis	Hipp_L_Ant	2.21	0.0431	0.39
R_Dorsal_boundary_Zaxis	Hipp_L_Ant_1	2.07	0.0431	0.37
R_CB36	Hipp_L_Ant_3_Rev2	1.99	0.0431	0.35
Hipp_L_Post3	R_CB38	2.62	0.0439	0.47
Hipp_L_Post3	R_CB18	2.51	0.0439	0.45

Hipp_L_Post3	R_CB36	2.35	0.0462	0.42
R_CB30	Hipp_L_Ant_1	2.06	0.0462	0.37
R_CB18	Hipp_L_Post3	2.51	0.0469	0.45
R_CB18	Hipp_L_Ant	2.23	0.0469	0.40
Hipp_L_Post3	R_CB35	2.18	0.0469	0.39
R_CB31	Hipp_L_Ant_2	2.38	0.0469	0.42
R_CB31	Hipp_L_Ant_1	2.06	0.0469	0.37
R_Medial_boundary_Xaxis	Hipp_L_Ant_1	2.65	0.0469	0.47
Hipp_L_Ant_3_Rev2	R_CB36	1.99	0.0469	0.35
Hipp_L_Ant_3_Rev2	R_Medial_boundary_Xaxis	1.98	0.0469	0.35
Hipp_L_Post1	R_CB37	-2.65	0.0474	-0.47
R_CB7	Hipp_L_Ant	2.22	0.0474	0.40
R_CB15	Hipp_L_Ant_2	2.03	0.0494	0.36
R_Medial_boundary_Xaxis	Hipp_L_Ant	2.14	0.0494	0.38
R_Medial_boundary_Xaxis	Hipp_L_Ant_3_Rev2	1.98	0.0494	0.35
R_CB14	Hipp_L_Ant_1	2.06	0.0494	0.37

Supplementary Table 5. Cerebellar-hippocampal regions showing significant correlations with increased progesterone levels.

FC Seed Regions		T(128)	pFDR	Cohen's d
R_CB37	Hipp_L_Post1	-6.43	0.0000	-1.14
R_CB36	Hipp_L_Post1	-6.14	0.0000	-1.09
Hipp_L_Post1	R_CB37	-6.43	0.0000	-1.14
R_Dorsal_boundary_Zaxis	Hipp_L_Ant_1	-5.81	0.0000	-1.03
Hipp_L_Post1	R_CB36	-6.14	0.0000	-1.09
R_CB30	Hipp_L_Post2	-5.92	0.0000	-1.05
R_CB38	Hipp_L_Post1	-5.82	0.0000	-1.03
Hipp_L_Post2	R_CB30	-5.92	0.0000	-1.05
Hipp_L_Ant_1	R_Dorsal_boundary_	-5.81	0.0000	-1.03
Hipp_L_Post1	R_CB38	-5.82	0.0000	-1.03
R_CB5	Hipp_L_Post1	-5.36	0.0000	-0.95
R_CB39	Hipp_L_Ant_1	-5.39	0.0000	-0.95
Hipp_L_Ant_1	R_CB39	-5.39	0.0000	-0.95
Hipp_L_Post1	R_CB5	-5.36	0.0000	-0.95
R_CB9	Hipp_L_Ant_1	-4.88	0.0000	-0.86
R_CB4	Hipp_L_Post1	-4.71	0.0000	-0.83
Hipp_L_Ant_1	R_CB9	-4.88	0.0000	-0.86
R_CB7	Hipp_L_Post2	-4.66	0.0000	-0.82
Hipp_L_Post1	R_CB4	-4.71	0.0000	-0.83
R_CB9	Hipp_L_Post3	-4.40	0.0000	-0.78
Hipp_L_Post2	R_CB7	-4.66	0.0000	-0.82
R_Dorsal_boundary_Zaxis	Hipp_L_Post3	-4.22	0.0000	-0.75
Hipp_L_Post3	R_CB9	-4.40	0.0000	-0.78

R_CB31	Hipp_L_Post2	-4.04	0.0001	-0.71
R_Rostral_boundary_Yaxis	Hipp_L_Post2	-4.28	0.0000	-0.76
Hipp_L_Post2	R_Rostral_boundary	-4.28	0.0000	-0.76
R_Dorsal_boundary_Zaxis	Hipp_L_Ant_2	-4.01	0.0001	-0.71
Hipp_L_Post3	R_Dorsal_boundary_	-4.22	0.0000	-0.75
Hipp_L_Post2	R_CB31	-4.04	0.0001	-0.71
R_Ventral_boundary_Zaxis	Hipp_L_Ant_1	3.98	0.0001	0.70
R_CB7	Hipp_L_Post1	-3.9	0.0002	-0.69
R_Dorsal_boundary_Zaxis	Hipp_L_Ant_3_Rev2	-3.77	0.0002	-0.67
R_Dorsal_boundary_Zaxis	Hipp_L_Post2	-3.73	0.0003	-0.66
Hipp_L_Ant_2	R_Dorsal_boundary_	-4.01	0.0001	-0.71
R_CB9	Hipp_L_Post1	-3.61	0.0004	-0.64
Hipp_L_Ant_1	R_Ventral_boundary	3.98	0.0001	0.70
Hipp_L_Post1	R_CB7	-3.90	0.0002	-0.69
R_CB19	Hipp_L_Post3	3.67	0.0003	0.65
R_Medial_boundary_Xaxis	Hipp_L_Ant_2	-3.59	0.0005	-0.63
R_CB39	Hipp_L_Ant_2	-3.64	0.0004	-0.64
Hipp_L_Post2	R_Dorsal_boundary_	-3.73	0.0003	-0.66
R_CB9	Hipp_L_Ant_3_Rev2	-3.46	0.0007	-0.61
R_CB9	Hipp_L_Ant_2	-3.46	0.0007	-0.61
R_CB26	Hipp_L_Post2	-3.51	0.0006	-0.62
R_CB39	Hipp_L_Ant_3_Rev2	-3.49	0.0007	-0.62
R_CB18	Hipp_L_Post1	-3.50	0.0006	-0.62
Hipp_L_Post3	R_CB19	3.67	0.0003	0.65
Hipp_L_Post1	R_CB9	-3.61	0.0004	-0.64
Hipp_L_Post2	R_CB26	-3.51	0.0006	-0.62
Hipp_L_Post1	R_CB18	-3.5	0.0006	-0.62
Hipp_L_Ant_2	R_CB39	-3.64	0.0004	-0.64
R_Ventral_boundary_Zaxis	Hipp_L_Ant_3_Rev2	3.46	0.0007	0.61
R_Caudal_boundary_Yaxis	Hipp_L_Post2	-3.29	0.0013	-0.58
Hipp_L_Ant_2	R_Medial_boundary_	-3.59	0.0005	-0.63
Hipp_L_Ant_3_Rev2	R_Dorsal_boundary_	-3.77	0.0002	-0.67
R_CB35	Hipp_L_Ant_3_Rev2	-3.21	0.0017	-0.57
R_CB19	Hipp_L_Post2	3.23	0.0016	0.57
Hipp_L_Ant_2	R_CB9	-3.46	0.0007	-0.61
R_Ventral_boundary_Zaxis	Hipp_L_Post3	3.27	0.0014	0.58
Hipp_L_Post2	R_Caudal_boundary_	-3.29	0.0013	-0.58
R_CB4	Hipp_L_Post2	-3.14	0.0021	-0.56
Hipp_L_Post2	R_CB19	3.23	0.0016	0.57
Hipp_L_Post3	R_Ventral_boundary	3.27	0.0014	0.58
Hipp_L_Ant_3_Rev2	R_CB39	-3.49	0.0007	-0.62
Hipp_L_Ant_3_Rev2	R_CB9	-3.46	0.0007	-0.61
Hipp_L_Ant_3_Rev2	R_Ventral_boundary	3.46	0.0007	0.61
Hipp_L_Post2	R_CB4	-3.14	0.0021	-0.56
R_Medial_boundary_Xaxis	Hipp_L_Post2	-2.97	0.0036	-0.53

R_Caudal_boundary_Yaxis	Hipp_L_Ant_1	-2.86	0.0050	-0.51
Hipp_L_Post2	R_Medial_boundary_	-2.97	0.0036	-0.53
Hipp_L_Ant_3_Rev2	R_CB35	-3.21	0.0017	-0.57
R_Dorsal_boundary_Zaxis	Hipp_L_Ant	-2.78	0.0063	-0.49
R_Medial_boundary_Xaxis	Hipp_L_Ant	-2.6	0.0105	-0.46
R_Caudal_boundary_Yaxis	Hipp_L_Post3	-2.59	0.0107	-0.46
R_CB37	Hipp_L_Ant_3_Rev2	-2.56	0.0116	-0.45
R_CB39	Hipp_L_Post3	-2.6	0.0103	-0.46
R_CB35	Hipp_L_Post1	-2.56	0.0116	-0.45
Hipp_L_Ant_1	R_Caudal_boundary_	-2.86	0.0050	-0.51
R_CB14	Hipp_L_Ant_1	-2.61	0.0102	-0.46
R_CB8	Hipp_L_Post1	-2.41	0.0175	-0.43
Hipp_L_Post1	R_CB35	-2.56	0.0116	-0.45
Hipp_L_Post3	R_CB39	-2.60	0.0103	-0.46
Hipp_L_Post3	R_Caudal_boundary_	-2.59	0.0107	-0.46
R_Medial_boundary_Xaxis	Hipp_L_Ant_3_Rev2	-2.32	0.0218	-0.41
Hipp_L_Ant_1	R_CB14	-2.61	0.0102	-0.46
R_CB30	Hipp_L_Post1	-2.39	0.0183	-0.42
R_CB18	Hipp_L_Ant_1	2.31	0.0225	0.41
R_CB15	Hipp_L_Post3	2.30	0.0233	0.41
R_Caudal_boundary_Yaxis	Hipp_L_Post1	-2.22	0.0280	-0.39
R_CB35	Hipp_L_Ant	-2.22	0.0281	-0.39
R_CB5	Hipp_L_Post2	-2.25	0.0263	-0.40
Hipp_L_Post1	R_CB8	-2.41	0.0175	-0.43
Hipp_L_Post1	R_CB30	-2.39	0.0183	-0.42
R_CB39	Hipp_L_Ant	-2.19	0.0302	-0.39
R_Caudal_boundary_Yaxis	Hipp_L_Ant	-2.14	0.0339	-0.38
R_CB36	Hipp_L_Post2	-2.16	0.0328	-0.38
R_CB19	Hipp_L_Post1	-2.23	0.0274	-0.39
R_Rostral_boundary_Yaxis	Hipp_L_Post1	-2.28	0.0243	-0.40
Hipp_L_Post1	R_Rostral_boundary	-2.28	0.0243	-0.40
Hipp_L_Ant	R_Dorsal_boundary_	-2.78	0.0063	-0.49
Hipp_L_Post1	R_CB19	-2.23	0.0274	-0.39
Hipp_L_Post1	R_Caudal_boundary_	-2.22	0.0280	-0.39
R_CB9	Hipp_L_Ant	-2.05	0.0425	-0.36
Hipp_L_Post2	R_CB5	-2.25	0.0263	-0.40
Hipp_L_Ant_3_Rev2	R_CB37	-2.56	0.0116	-0.45
Hipp_L_Post3	R_CB15	2.30	0.0233	0.41
R_Medial_boundary_Xaxis	Hipp_L_Ant_1	-2.02	0.0456	-0.36
R_CB26	Hipp_L_Post1	-2.00	0.0478	-0.35
R_CB8	Hipp_L_Post3	-2.01	0.0461	-0.36
Hipp_L_Post2	R_CB36	-2.16	0.0328	-0.38
Hipp_L_Ant	R_Medial_boundary_	-2.60	0.0105	-0.46
Hipp_L_Ant_1	R_CB18	2.31	0.0225	0.41
R_Ventral_boundary_Zaxis	Hipp_L_Ant_2	2.05	0.0428	0.36

R_CB7	Hipp_L_Ant_1	-2.05	0.0424	-0.36
R_CB19	Hipp_L_Ant_2	-1.99	0.0492	-0.35
Hipp_L_Post1	R_CB26	-2.00	0.0478	-0.35
Hipp_L_Ant_3_Rev2	R_Medial_boundary_	-2.32	0.0218	-0.41
Hipp_L_Post3	R_CB8	-2.01	0.0461	-0.36
Hipp_L_Ant_1	R_CB7	-2.05	0.0424	-0.36
Hipp_L_Ant_1	R_Medial_boundary_	-2.02	0.0456	-0.36
Hipp_L_Ant_2	R_Ventral_boundary	2.05	0.0428	0.36
Hipp_L_Ant_2	R_CB19	-1.99	0.0492	-0.35
Hipp_L_Ant	R_CB35	-2.22	0.0281	-0.39
Hipp_L_Ant	R_CB39	-2.19	0.0302	-0.39
Hipp_L_Ant	R_Caudal_boundary_	-2.14	0.0339	-0.38
Hipp_L_Ant	R_CB9	-2.05	0.0425	-0.36
R_CB32	Hipp_L_Post2	-5.77	0.0000	-1.02
R_CB29	Hipp_L_Post3	-5.81	0.0000	-1.03
Hipp_L_Post3	R_CB29	-5.81	0.0000	-1.03
Hipp_L_Post2	R_CB32	-5.77	0.0000	-1.02
R_CB33	Hipp_L_Post3	-5.28	0.0000	-0.93
R_CB33	Hipp_L_Ant_1	-5.22	0.0000	-0.92
R_CB29	Hipp_L_Post2	-5.15	0.0000	-0.91
Hipp_L_Post3	R_CB33	-5.28	0.0000	-0.93
R_CB2	Hipp_L_Post3	-4.99	0.0000	-0.88
Hipp_L_Ant_1	R_CB33	-5.22	0.0000	-0.92
Hipp_L_Post2	R_CB29	-5.15	0.0000	-0.91
R_CB6	Hipp_L_Post1	-4.73	0.0000	-0.84
R_CB33	Hipp_L_Post2	-4.76	0.0000	-0.84
R_CB27	Hipp_L_Post2	-4.66	0.0000	-0.82
Hipp_L_Post3	R_CB2	-4.99	0.0000	-0.88
R_CB27	Hipp_L_Ant_2	-4.59	0.0000	-0.81
R_CB2	Hipp_L_Ant_1	-4.64	0.0000	-0.82
R_CB32	Hipp_L_Ant_2	-4.54	0.0000	-0.80
Hipp_L_Post2	R_CB33	-4.76	0.0000	-0.84
Hipp_L_Post1	R_CB6	-4.73	0.0000	-0.84
R_CB2	Hipp_L_Post1	-4.46	0.0000	-0.79
Hipp_L_Post2	R_CB27	-4.66	0.0000	-0.82
Hipp_L_Ant_1	R_CB2	-4.64	0.0000	-0.82
R_CB22	Hipp_L_Post2	-4.28	0.0000	-0.76
R_CB27	Hipp_L_Post1	-4.17	0.0001	-0.74
Hipp_L_Post1	R_CB2	-4.46	0.0000	-0.79
Hipp_L_Ant_2	R_CB27	-4.59	0.0000	-0.81
Hipp_L_Ant_2	R_CB32	-4.54	0.0000	-0.80
R_CB22	Hipp_L_Ant_2	-4.14	0.0001	-0.73
Hipp_L_Post2	R_CB22	-4.28	0.0000	-0.76
R_CB32	Hipp_L_Ant_1	-3.97	0.0001	-0.70
R_CB23	Hipp_L_Ant_2	-4.04	0.0001	-0.71

R_CB22	Hipp_L_Post1	-3.97	0.0001	-0.70
Hipp_L_Post1	R_CB27	-4.17	0.0001	-0.74
R_CB27	Hipp_L_Post3	-3.84	0.0002	-0.68
R_CB28	Hipp_L_Post2	-3.78	0.0002	-0.67
R_CB23	Hipp_L_Post1	-3.82	0.0002	-0.68
Hipp_L_Ant_2	R_CB22	-4.14	0.0001	-0.73
Hipp_L_Post1	R_CB22	-3.97	0.0001	-0.70
R_CB29	Hipp_L_Post1	-3.69	0.0003	-0.65
Hipp_L_Ant_2	R_CB23	-4.04	0.0001	-0.71
Hipp_L_Ant_1	R_CB32	-3.97	0.0001	-0.70
R_CB29	Hipp_L_Ant_1	-3.57	0.0005	-0.63
Hipp_L_Post1	R_CB23	-3.82	0.0002	-0.68
Hipp_L_Post2	R_CB28	-3.78	0.0002	-0.67
R_CB16	Hipp_L_Post1	-3.55	0.0005	-0.63
Hipp_L_Post3	R_CB27	-3.84	0.0002	-0.68
R_CB21	Hipp_L_Post1	-3.55	0.0005	-0.63
Hipp_L_Post1	R_CB29	-3.69	0.0003	-0.65
R_CB28	Hipp_L_Post1	-3.38	0.0010	-0.60
R_CB13	Hipp_L_Post1	-3.42	0.0008	-0.60
R_CB28	Hipp_L_Ant_1	-3.34	0.0011	-0.59
Hipp_L_Post1	R_CB21	-3.55	0.0005	-0.63
Hipp_L_Post1	R_CB16	-3.55	0.0005	-0.63
R_CB24	Hipp_L_Post3	-3.31	0.0012	-0.59
Hipp_L_Post1	R_CB13	-3.42	0.0008	-0.60
Hipp_L_Ant_1	R_CB29	-3.57	0.0005	-0.63
Hipp_L_Post1	R_CB28	-3.38	0.0010	-0.60
R_CB17	Hipp_L_Post3	3.27	0.0014	0.58
R_CB22	Hipp_L_Ant_1	-3.15	0.0020	-0.56
R_CB23	Hipp_L_Post3	-3.19	0.0018	-0.56
R_CB28	Hipp_L_Ant_2	-3.12	0.0022	-0.55
R_CB24	Hipp_L_Post1	-3.13	0.0022	-0.55
R_CB23	Hipp_L_Post2	-3.1	0.0024	-0.55
R_CB22	Hipp_L_Post3	-3.01	0.0032	-0.53
Hipp_L_Post3	R_CB24	-3.31	0.0012	-0.59
Hipp_L_Post3	R_CB17	3.27	0.0014	0.58
Hipp_L_Ant_1	R_CB28	-3.34	0.0011	-0.59
Hipp_L_Post1	R_CB24	-3.13	0.0022	-0.55
R_CB2	Hipp_L_Ant	-2.98	0.0035	-0.53
Hipp_L_Post3	R_CB23	-3.19	0.0018	-0.56
Hipp_L_Post2	R_CB23	-3.1	0.0024	-0.55
Hipp_L_Ant_1	R_CB22	-3.15	0.0020	-0.56
Hipp_L_Ant_2	R_CB28	-3.12	0.0022	-0.55
R_CB33	Hipp_L_Ant_3_Rev2	-2.75	0.0068	-0.49
Hipp_L_Post3	R_CB22	-3.01	0.0032	-0.53
R_CB27	Hipp_L_Ant_1	-2.67	0.0085	-0.47

R_CB28	Hipp_L_Ant	-2.67	0.0086	-0.47
R_CB2	Hipp_L_Post2	-2.72	0.0075	-0.48
R_CB6	Hipp_L_Ant_2	-2.68	0.0083	-0.47
R_CB33	Hipp_L_Ant_2	-2.58	0.0110	-0.46
R_CB2	Hipp_L_Ant_2	-2.58	0.0111	-0.46
Hipp_L_Post2	R_CB2	-2.72	0.0075	-0.48
R_CB24	Hipp_L_Post2	-2.5	0.0138	-0.44
R_CB32	Hipp_L_Post1	-2.41	0.0174	-0.43
R_CB28	Hipp_L_Ant_3_Rev2	-2.36	0.0199	-0.42
Hipp_L_Ant_1	R_CB27	-2.67	0.0085	-0.47
Hipp_L_Post2	R_CB24	-2.5	0.0138	-0.44
Hipp_L_Ant_2	R_CB6	-2.68	0.0083	-0.47
R_CB32	Hipp_L_Ant_3_Rev2	-2.26	0.0257	-0.40
Hipp_L_Ant	R_CB2	-2.98	0.0035	-0.53
R_CB28	Hipp_L_Post3	-2.21	0.0287	-0.39
Hipp_L_Ant_2	R_CB33	-2.58	0.0110	-0.46
Hipp_L_Ant_2	R_CB2	-2.58	0.0111	-0.46
Hipp_L_Ant_3_Rev2	R_CB33	-2.75	0.0068	-0.49
R_Lateral_boundary_Xaxis	Hipp_L_Ant_2	-2.3	0.0232	-0.41
Hipp_L_Post1	R_CB32	-2.41	0.0174	-0.43
R_CB32	Hipp_L_Post3	-2.1	0.0378	-0.37
R_CB17	Hipp_L_Post1	-2.18	0.0313	-0.39
R_CB17	Hipp_L_Ant_3_Rev2	2.13	0.0351	0.38
R_Lateral_boundary_Xaxis	Hipp_L_Ant_1	-2.07	0.0401	-0.37
Hipp_L_Post1	R_CB17	-2.18	0.0313	-0.39
R_CB21	Hipp_L_Post3	2.06	0.0414	0.36
Hipp_L_Ant	R_CB28	-2.67	0.0086	-0.47
R_CB25	Hipp_L_Post3	-2.1	0.0377	-0.37
Hipp_L_Post3	R_CB28	-2.21	0.0287	-0.39
R_CB25	Hipp_L_Post2	2.04	0.0431	0.36
Hipp_L_Ant_2	R_Lateral_boundary	-2.3	0.0232	-0.41
Hipp_L_Post2	R_CB25	2.04	0.0431	0.36
Hipp_L_Post3	R_CB25	-2.1	0.0377	-0.37
Hipp_L_Post3	R_CB32	-2.1	0.0378	-0.37
Hipp_L_Ant_3_Rev2	R_CB28	-2.36	0.0199	-0.42
Hipp_L_Post3	R_CB21	2.06	0.0414	0.36
Hipp_L_Ant_3_Rev2	R_CB32	-2.26	0.0257	-0.40
Hipp_L_Ant_1	R_Lateral_boundary	-2.07	0.0401	-0.37
Hipp_L_Ant_3_Rev2	R_CB17	2.13	0.0351	0.38
R_CB11	Hipp_L_Post1	-5.2	0.0000	-0.92
R_CB1	Hipp_L_Post1	-5.2	0.0000	-0.92
Hipp_L_Post1	R_CB11	-5.2	0.0000	-0.92
Hipp_L_Post1	R_CB1	-5.2	0.0000	-0.92
R_CB1	Hipp_L_Post2	-4.09	0.0001	-0.72
R_CB11	Hipp_L_Post2	-4.09	0.0001	-0.72

Hipp_L_Post2	R_CB11	-4.09	0.0001	-0.72
Hipp_L_Post2	R_CB1	-4.09	0.0001	-0.72
R_CB11	Hipp_L_Post3	-3.67	0.0004	-0.65
R_CB1	Hipp_L_Post3	-3.67	0.0004	-0.65
Hipp_L_Post3	R_CB11	-3.67	0.0004	-0.65
Hipp_L_Post3	R_CB1	-3.67	0.0004	-0.65
R_CB11	Hipp_L_Ant_1	-2	0.0478	-0.35
R_CB1	Hipp_L_Ant_1	-2	0.0478	-0.35
Hipp_L_Ant_1	R_CB11	-2	0.0478	-0.35
Hipp_L_Ant_1	R_CB1	-2	0.0478	-0.35

Supplementary Table 6. Intra-cerebellar and intra-hippocampal regions showing significant correlations with increased progesterone levels.

FC Seed Regions		T(128)	pFDR	Cohen's d
R_CB1	R_CB11	314.77	0.0000	55.6440
R_CB11	R_CB1	311.98	0.0000	55.1508
R_CB23	R_CB22	25.6	0.0000	4.5255
R_CB22	R_CB23	25.6	0.0000	4.5255
R_CB24	R_CB23	24.84	0.0000	4.3911
R_CB23	R_CB24	24.84	0.0000	4.3911
R_CB12	R_CB13	23.25	0.0000	4.1101
R_CB13	R_CB12	23.25	0.0000	4.1101
R_CB13	R_CB16	23.21	0.0000	4.1030
R_CB16	R_CB13	23.21	0.0000	4.1030
R_CB24	R_CB24	22.43	0.0000	3.9651
R_CB22	R_CB36	22.3	0.0000	3.9421
R_CB27	R_CB28	22.18	0.0000	3.9209
R_CB28	R_CB27	22.18	0.0000	3.9209
R_CB24	R_CB28	21.98	0.0000	3.8856
R_CB28	R_CB24	21.98	0.0000	3.8856
R_CB13	R_CB24	21.15	0.0000	3.7388
R_CB24	R_CB13	21.15	0.0000	3.7388
R_CB32	R_CB23	21	0.0000	3.7123
R_CB33	R_CB23	20.18	0.0000	3.5674
R_CB13	R_CB12	20	0.0000	3.5355
R_CB23	R_CB27	19.87	0.0000	3.5126
R_CB28	R_CB23	19.35	0.0000	3.4206
R_CB23	R_CB12	19.35	0.0000	3.4206
R_CB12	R_CB20	19.23	0.0000	3.3994
R_CB27	R_CB16	19.23	0.0000	3.3994
R_CB12	R_CB36	19.15	0.0000	3.3853
R_CB23	R_CB28	19.14	0.0000	3.3835

R_CB16	R_CB29	19.14	0.0000	3.3835
R_CB20	R_CB27	19.1	0.0000	3.3764
R_CB29	R_CB24	19.1	0.0000	3.3764
R_CB28	R_CB32	19.01	0.0000	3.3605
R_CB24	R_CB28	19.01	0.0000	3.3605
R_CB27	R_CB21	18.34	0.0000	3.2421
R_CB32	R_CB17	18.34	0.0000	3.2421
R_CB28	R_CB27	18.26	0.0000	3.2279
R_CB17	R_CB27	18.26	0.0000	3.2279
R_CB21	R_CB32	18.26	0.0000	3.2279
R_CB32	R_CB31	18.26	0.0000	3.2279
R_CB27	R_CB29	18.17	0.0000	3.2120
R_CB29	R_CB32	18.17	0.0000	3.2120
R_CB17	R_CB32	18.08	0.0000	3.1961
R_CB16	R_CB8	17.9	0.0000	3.1643
R_CB23	R_CB5	17.82	0.0000	3.1502
R_CB22	R_CB38	17.58	0.0000	3.1077
R_CB28	R_CB35	17.58	0.0000	3.1077
R_CB13	R_CB28	17.19	0.0000	3.0388
R_CB22	R_CB22	17.19	0.0000	3.0388
R_CB29	R_CB22	16.98	0.0000	3.0017
R_CB24	R_CB13	16.98	0.0000	3.0017
R_CB29	R_CB29	16.96	0.0000	2.9981
R_CB13	R_CB27	16.96	0.0000	2.9981
R_CB17	R_CB37	16.91	0.0000	2.9893
R_CB2	R_CB27	16.7	0.0000	2.9522
R_CB3	R_CB26	16.69	0.0000	2.9504
R_CB21	R_CB31	16.2	0.0000	2.8638
R_Lateral_boundary_Xaxis	R_CB28	16.2	0.0000	2.8638
R_CB23	R_CB13	16.14	0.0000	2.8532
R_CB29	R_CB17	16.14	0.0000	2.8532
R_CB16	R_CB3	16.12	0.0000	2.8496
R_CB27	R_Lateral_boundary_Xaxis	16.03	0.0000	2.8337
R_CB21	R_CB21	16.03	0.0000	2.8337
R_CB20	R_CB8	15.88	0.0000	2.8072
R_CB24	R_CB9	15.88	0.0000	2.8072
R_CB16	R_CB37	15.79	0.0000	2.7913
R_CB29	R_CB23	15.75	0.0000	2.7842
R_CB33	R_CB27	15.65	0.0000	2.7666
R_CB12	R_CB16	15.65	0.0000	2.7666
R_CB12	R_CB20	15.63	0.0000	2.7630
R_CB16	R_CB21	15.63	0.0000	2.7630
R_CB16	R_CB35	15.59	0.0000	2.7559
R_CB17	R_CB16	15.58	0.0000	2.7542
R_CB28	R_CB29	15.52	0.0000	2.7436

R_CB29	R_CB15	15.48	0.0000	2.7365
R_CB22	R_CB22	15.44	0.0000	2.7294
R_CB25	R_CB4	15.42	0.0000	2.7259
R_CB3	R_CB12	15.27	0.0000	2.6994
R_CB28	R_CB7	15.25	0.0000	2.6958
R_CB20	R_CB8	15.25	0.0000	2.6958
R_CB17	R_CB28	15.14	0.0000	2.6764
R_Lateral_boundary_Xaxis	R_CB16	15.14	0.0000	2.6764
R_CB17	R_CB11	15.1	0.0000	2.6693
R_CB28	R_CB2	15.1	0.0000	2.6693
R_CB6	R_CB2	15.1	0.0000	2.6693
R_CB24	R_CB17	14.97	0.0000	2.6463
R_CB25	R_CB31	14.72	0.0000	2.6022
R_CB25	R_CB9	14.67	0.0000	2.5933
R_CB17	R_CB15	14.65	0.0000	2.5898
R_CB12	R_CB12	14.65	0.0000	2.5898
R_CB27	R_CB9	14.58	0.0000	2.5774
R_CB16	R_CB33	14.58	0.0000	2.5774
R_CB21	R_CB22	14.48	0.0000	2.5597
R_CB13	R_CB29	14.48	0.0000	2.5597
R_CB28	R_CB25	14.47	0.0000	2.5580
R_CB29	R_CB31	14.22	0.0000	2.5138
R_CB6	R_CB28	14.19	0.0000	2.5085
R_CB17	R_CB17	14.09	0.0000	2.4908
R_CB17	R_CB20	14.09	0.0000	2.4908
R_Lateral_boundary_Xaxis	R_CB29	14.08	0.0000	2.4890
R_CB16	R_CB31	13.96	0.0000	2.4678
R_CB22	R_Dorsal_boundary_Zaxis	13.96	0.0000	2.4678
R_CB29	R_CB17	13.88	0.0000	2.4537
R_CB20	R_CB6	13.82	0.0000	2.4431
R_CB23	R_CB6	13.82	0.0000	2.4431
R_CB32	R_CB15	13.72	0.0000	2.4254
R_CB13	R_CB19	13.72	0.0000	2.4254
R_CB25	R_CB6	13.69	0.0000	2.4201
R_CB28	R_CB5	13.69	0.0000	2.4201
R_CB33	R_CB25	13.58	0.0000	2.4006
R_CB27	R_CB24	13.58	0.0000	2.4006
R_CB13	R_CB17	13.49	0.0000	2.3847
R_CB28	R_CB25	13.49	0.0000	2.3847
R_CB25	R_CB21	13.28	0.0000	2.3476
R_CB27	R_CB26	13.28	0.0000	2.3476
R_CB33	R_CB16	13.28	0.0000	2.3476
R_CB29	R_CB7	13.26	0.0000	2.3441
R_CB16	R_CB4	13.26	0.0000	2.3441
R_CB6	R_CB15	13.22	0.0000	2.3370

R_CB29	R_CB29	13.21	0.0000	2.3352
R_CB12	R_CB31	13.21	0.0000	2.3352
R_CB10	R_CB13	13.21	0.0000	2.3352
R_CB16	R_CB28	13.21	0.0000	2.3352
R_CB28	R_CB23	13.09	0.0000	2.3140
R_CB12	R_CB26	13.09	0.0000	2.3140
R_CB16	R_CB6	13.09	0.0000	2.3140
R_CB25	R_CB18	13.09	0.0000	2.3140
R_CB22	R_CB14	13.09	0.0000	2.3140
R_CB6	R_CB29	13.09	0.0000	2.3140
R_CB28	R_CB17	13.09	0.0000	2.3140
R_Lateral_boundary_Xaxis	R_CB30	13.09	0.0000	2.3140
R_CB6	R_CB16	12.99	0.0000	2.2963
R_CB2	R_CB17	12.99	0.0000	2.2963
R_CB23	R_CB29	12.89	0.0000	2.2787
R_CB25	R_CB26	12.85	0.0000	2.2716
R_CB13	R_CB30	12.83	0.0000	2.2680
R_CB20	R_CB27	12.83	0.0000	2.2680
R_CB29	R_CB13	12.79	0.0000	2.2610
R_CB21	R_CB23	12.79	0.0000	2.2610
R_CB29	R_CB32	12.79	0.0000	2.2610
R_CB25	R_CB13	12.74	0.0000	2.2521
R_CB25	R_CB25	12.7	0.0000	2.2451
R_Lateral_boundary_Xaxis	R_CB26	12.68	0.0000	2.2415
R_CB20	R_CB33	12.63	0.0000	2.2327
R_CB28	R_CB27	12.63	0.0000	2.2327
R_CB20	R_CB4	12.61	0.0000	2.2292
R_CB29	R_CB29	12.53	0.0000	2.2150
R_CB17	R_CB16	12.51	0.0000	2.2115
R_CB21	R_CB6	12.51	0.0000	2.2115
R_CB20	R_CB9	12.51	0.0000	2.2115
R_Lateral_boundary_Xaxis	R_CB9	12.48	0.0000	2.2062
R_CB32	R_Dorsal_boundary_Zaxis	12.46	0.0000	2.2026
R_CB22	R_CB39	12.46	0.0000	2.2026
R_CB25	R_CB20	12.37	0.0000	2.1867
R_CB24	R_CB15	12.37	0.0000	2.1867
R_CB6	R_CB12	12.33	0.0000	2.1797
R_CB17	R_CB21	12.23	0.0000	2.1620
R_CB12	R_CB10	12.23	0.0000	2.1620
R_CB27	R_CB13	12.18	0.0000	2.1531
R_CB2	R_CB28	12.18	0.0000	2.1531
R_CB27	R_CB24	12.12	0.0000	2.1425
R_CB20	R_CB25	12.11	0.0000	2.1408
R_CB27	R_CB16	12.11	0.0000	2.1408
R_CB6	R_CB22	12.07	0.0000	2.1337

R_CB32	R_CB13	12.01	0.0000	2.1231
R_CB21	R_CB15	12.01	0.0000	2.1231
R_CB24	R_CB28	11.96	0.0000	2.1142
R_CB23	R_CB6	11.92	0.0000	2.1072
R_CB21	R_CB2	11.92	0.0000	2.1072
R_CB20	R_CB22	11.88	0.0000	2.1001
R_CB33	R_CB25	11.77	0.0000	2.0807
R_CB13	R_CB23	11.77	0.0000	2.0807
R_CB32	R_CB20	11.6	0.0000	2.0506
R_CB6	R_CB13	11.6	0.0000	2.0506
R_CB12	R_CB19	11.55	0.0000	2.0418
R_CB6	R_CB16	11.55	0.0000	2.0418
R_CB32	R_CB22	11.52	0.0000	2.0365
R_Lateral_boundary_Xaxis	R_CB21	11.52	0.0000	2.0365
R_CB28	R_CB29	11.51	0.0000	2.0347
R_CB3	R_CB25	11.51	0.0000	2.0347
R_CB22	R_Lateral_boundary_Xaxis	11.5	0.0000	2.0329
R_CB3	R_CB20	11.46	0.0000	2.0259
R_CB6	R_CB12	11.46	0.0000	2.0259
R_CB21	R_CB28	11.42	0.0000	2.0188
R_CB23	R_CB20	11.42	0.0000	2.0188
R_CB13	R_CB17	11.42	0.0000	2.0188
R_CB3	R_CB29	11.42	0.0000	2.0188
R_CB32	R_CB21	11.39	0.0000	2.0135
R_CB13	R_CB24	11.39	0.0000	2.0135
R_Lateral_boundary_Xaxis	R_Ventral_boundary_Zaxis	11.37	0.0000	2.0100
R_CB28	R_CB17	11.37	0.0000	2.0100
R_CB2	R_Lateral_boundary_Xaxis	11.33	0.0000	2.0029
R_CB20	R_CB20	11.33	0.0000	2.0029
R_CB16	R_CB32	11.26	0.0000	1.9905
R_Lateral_boundary_Xaxis	R_CB20	11.26	0.0000	1.9905
R_CB20	R_CB26	11.22	0.0000	1.9834
R_CB10	R_CB24	11.22	0.0000	1.9834
R_Lateral_boundary_Xaxis	R_CB25	11.19	0.0000	1.9781
R_CB33	R_CB38	11.17	0.0000	1.9746
R_CB22	R_CB24	11.16	0.0000	1.9728
R_CB2	R_CB35	11.1	0.0000	1.9622
R_CB16	R_CB4	11.1	0.0000	1.9622
R_CB2	R_CB17	11.07	0.0000	1.9569
R_CB12	R_CB27	11.07	0.0000	1.9569
R_Lateral_boundary_Xaxis	R_CB12	11.07	0.0000	1.9569
R_CB23	R_CB2	11.07	0.0000	1.9569
R_Lateral_boundary_Xaxis	R_CB20	10.91	0.0000	1.9286
R_CB27	R_CB36	10.9	0.0000	1.9269
R_CB25	R_CB4	10.9	0.0000	1.9269

R_CB27	R_CB27	10.89	0.0000	1.9251
R_CB10	R_CB6	10.89	0.0000	1.9251
R_CB17	R_CB19	10.84	0.0000	1.9163
R_CB10	R_CB21	10.81	0.0000	1.9110
R_CB12	R_CB32	10.81	0.0000	1.9110
R_CB23	R_CB20	10.77	0.0000	1.9039
R_CB33	R_CB24	10.77	0.0000	1.9039
R_CB33	R_CB23	10.66	0.0000	1.8844
R_CB10	R_CB21	10.66	0.0000	1.8844
R_CB10	R_CB5	10.66	0.0000	1.8844
R_CB6	R_Caudal_boundary_Yaxis	10.66	0.0000	1.8844
R_CB17	R_CB18	10.39	0.0000	1.8367
R_CB20	R_CB15	10.39	0.0000	1.8367
R_CB22	R_CB33	10.35	0.0000	1.8296
R_CB6	R_CB20	10.35	0.0000	1.8296
R_CB13	R_CB4	10.3	0.0000	1.8208
R_CB24	R_CB1	10.3	0.0000	1.8208
R_CB10	R_CB9	10.29	0.0000	1.8190
R_CB24	R_CB20	10.29	0.0000	1.8190
R_CB32	R_CB9	10.26	0.0000	1.8137
R_CB2	R_CB27	10.26	0.0000	1.8137
R_CB21	R_CB22	10.23	0.0000	1.8084
R_CB33	R_CB31	10.23	0.0000	1.8084
R_CB12	R_CB13	10.23	0.0000	1.8084
R_CB25	R_CB6	10.21	0.0000	1.8049
R_Lateral_boundary_Xaxis	R_CB32	10.21	0.0000	1.8049
R_CB10	R_CB35	10.18	0.0000	1.7996
R_CB22	R_Lateral_boundary_Xaxis	10.17	0.0000	1.7978
R_CB21	R_CB12	10.17	0.0000	1.7978
R_CB10	R_CB32	10.17	0.0000	1.7978
R_CB16	R_Caudal_boundary_Yaxis	10.15	0.0000	1.7943
R_CB10	R_CB4	10.15	0.0000	1.7943
R_CB16	R_CB3	10.11	0.0000	1.7872
R_CB32	R_CB3	10.1	0.0000	1.7854
R_CB27	R_CB22	10.1	0.0000	1.7854
R_Lateral_boundary_Xaxis	R_CB23	9.95	0.0000	1.7589
R_CB13	R_CB6	9.95	0.0000	1.7589
R_CB2	R_CB3	9.88	0.0000	1.7466
R_CB21	R_CB32	9.86	0.0000	1.7430
R_CB25	R_CB1	9.84	0.0000	1.7395
R_CB20	R_CB13	9.79	0.0000	1.7306
R_CB29	R_Lateral_boundary_Xaxis	9.79	0.0000	1.7306
R_CB10	R_CB28	9.77	0.0000	1.7271
R_CB24	R_CB2	9.77	0.0000	1.7271
R_CB2	R_CB35	9.73	0.0000	1.7200

R_CB3	R_CB8	9.73	0.0000	1.7200
R_CB25	R_CB13	9.72	0.0000	1.7183
R_CB22	R_Ventral_boundary_Zaxis	9.72	0.0000	1.7183
R_CB10	R_CB9	9.71	0.0000	1.7165
R_CB12	R_CB7	9.71	0.0000	1.7165
R_CB28	R_CB5	9.71	0.0000	1.7165
R_CB10	R_CB7	9.71	0.0000	1.7165
R_CB10	R_CB36	9.68	0.0000	1.7112
R_CB13	R_Caudal_boundary_Yaxis	9.68	0.0000	1.7112
R_CB10	R_CB20	9.66	0.0000	1.7077
R_CB32	R_CB6	9.66	0.0000	1.7077
R_CB24	R_CB16	9.65	0.0000	1.7059
R_CB33	R_CB20	9.63	0.0000	1.7024
R_CB6	R_CB31	9.63	0.0000	1.7024
R_CB25	R_CB10	9.63	0.0000	1.7024
R_CB2	R_CB9	9.63	0.0000	1.7024
R_CB32	R_CB29	9.63	0.0000	1.7024
R_CB13	R_CB2	9.58	0.0000	1.6935
R_CB22	R_Caudal_boundary_Yaxis	9.56	0.0000	1.6900
R_CB23	R_CB36	9.55	0.0000	1.6882
R_CB2	R_CB8	9.55	0.0000	1.6882
R_Lateral_boundary_Xaxis	R_Rostral_boundary_Yaxis	9.47	0.0000	1.6741
R_CB29	R_CB30	9.47	0.0000	1.6741
R_CB2	R_CB9	9.47	0.0000	1.6741
R_CB3	R_Caudal_boundary_Yaxis	9.41	0.0000	1.6635
R_CB32	R_CB24	9.4	0.0000	1.6617
R_CB6	R_Ventral_boundary_Zaxis	9.4	0.0000	1.6617
R_CB16	R_CB23	9.38	0.0000	1.6582
R_CB6	R_Lateral_boundary_Xaxis	9.38	0.0000	1.6582
R_Lateral_boundary_Xaxis	R_CB25	9.35	0.0000	1.6529
R_CB33	R_CB27	9.35	0.0000	1.6529
R_CB25	R_CB16	9.29	0.0000	1.6423
R_CB33	R_CB2	9.29	0.0000	1.6423
R_CB21	R_CB5	9.29	0.0000	1.6423
R_CB21	R_CB3	9.29	0.0000	1.6423
R_CB3	R_CB6	9.26	0.0000	1.6370
R_CB25	R_CB38	9.26	0.0000	1.6370
R_CB10	R_CB5	9.26	0.0000	1.6370
R_CB20	R_CB3	9.26	0.0000	1.6370
R_CB2	R_CB10	9.24	0.0000	1.6334
R_CB2	R_CB3	9.24	0.0000	1.6334
R_CB10	R_Ventral_boundary_Zaxis	9.24	0.0000	1.6334
R_CB29	R_CB16	9.24	0.0000	1.6334
R_CB3	R_CB15	9.24	0.0000	1.6334
R_CB3	R_CB17	9.24	0.0000	1.6334

R_CB33	R_CB39	9.23	0.0000	1.6316
R_CB2	R_CB35	9.23	0.0000	1.6316
R_CB13	R_CB17	9.21	0.0000	1.6281
R_CB2	R_CB21	9.21	0.0000	1.6281
R_CB17	R_CB10	9.21	0.0000	1.6281
R_CB20	R_CB2	9.2	0.0000	1.6263
R_CB3	R_CB12	9.2	0.0000	1.6263
R_CB25	R_CB6	9.19	0.0000	1.6246
R_CB3	R_CB23	9.19	0.0000	1.6246
R_CB17	R_CB33	9.17	0.0000	1.6210
R_CB10	R_CB10	9.17	0.0000	1.6210
R_CB2	R_CB32	9.15	0.0000	1.6175
R_Lateral_boundary_Xaxis	R_CB32	9.15	0.0000	1.6175
R_CB3	R_CB5	9.15	0.0000	1.6175
R_CB38	R_CB27	9.14	0.0000	1.6157
R_CB36	R_Dorsal_boundary_Zaxis	9.14	0.0000	1.6157
R_CB36	R_CB23	9.13	0.0000	1.6140
R_CB35	R_Medial_boundary_Xaxis	9.12	0.0000	1.6122
R_CB8	R_CB39	9.12	0.0000	1.6122
R_CB4	R_CB4	9.11	0.0000	1.6104
R_CB8	R_CB5	9.1	0.0000	1.6087
R_CB5	R_CB17	9.09	0.0000	1.6069
R_CB35	R_CB14	9.09	0.0000	1.6069
R_CB38	R_CB19	9.09	0.0000	1.6069
R_CB37	R_Caudal_boundary_Yaxis	9.03	0.0000	1.5963
R_CB38	R_CB22	9.02	0.0000	1.5945
R_CB9	R_CB20	9.02	0.0000	1.5945
R_CB8	R_CB28	8.99	0.0000	1.5892
R_CB37	R_Dorsal_boundary_Zaxis	8.99	0.0000	1.5892
R_CB36	R_CB9	8.97	0.0000	1.5857
R_CB37	R_CB12	8.97	0.0000	1.5857
R_CB35	R_CB8	8.94	0.0000	1.5804
R_CB4	R_CB10	8.94	0.0000	1.5804
R_CB5	R_CB22	8.9	0.0000	1.5733
R_CB8	R_CB11	8.9	0.0000	1.5733
R_CB7	R_CB36	8.9	0.0000	1.5733
R_CB31	R_Ventral_boundary_Zaxis	8.9	0.0000	1.5733
R_CB30	R_CB5	8.89	0.0000	1.5715
R_CB31	R_CB22	8.89	0.0000	1.5715
R_Dorsal_boundary_Zaxis	R_CB30	8.88	0.0000	1.5698
R_CB19	R_Dorsal_boundary_Zaxis	8.88	0.0000	1.5698
R_CB15	R_Lateral_boundary_Xaxis	8.8	0.0000	1.5556
R_CB4	R_Ventral_boundary_Zaxis	8.76	0.0000	1.5486
R_CB7	R_Caudal_boundary_Yaxis	8.75	0.0000	1.5468
R_CB15	R_CB2	8.74	0.0000	1.5450

R_CB14	R_CB21	8.74	0.0000	1.5450
R_CB14	R_CB33	8.74	0.0000	1.5450
R_CB18	R_CB12	8.72	0.0000	1.5415
R_CB26	R_CB36	8.69	0.0000	1.5362
R_CB30	R_CB5	8.69	0.0000	1.5362
R_CB38	R_CB10	8.67	0.0000	1.5327
R_CB9	R_Lateral_boundary_Xaxis	8.67	0.0000	1.5327
R_CB5	R_CB21	8.66	0.0000	1.5309
R_Dorsal_boundary_Zaxis	R_CB35	8.66	0.0000	1.5309
R_CB39	R_CB22	8.66	0.0000	1.5309
R_CB31	R_CB30	8.63	0.0000	1.5256
R_CB26	R_CB23	8.62	0.0000	1.5238
R_CB8	R_Medial_boundary_Xaxis	8.58	0.0000	1.5167
R_CB38	R_CB23	8.54	0.0000	1.5097
R_CB4	R_CB11	8.53	0.0000	1.5079
R_CB35	R_CB3	8.53	0.0000	1.5079
R_CB4	R_Dorsal_boundary_Zaxis	8.52	0.0000	1.5061
R_CB36	R_CB36	8.52	0.0000	1.5061
R_CB19	R_CB28	8.48	0.0000	1.4991
R_CB18	R_CB35	8.46	0.0000	1.4955
R_CB5	R_CB33	8.46	0.0000	1.4955
R_Caudal_boundary_Yaxis	R_CB16	8.45	0.0000	1.4938
R_CB15	R_CB31	8.45	0.0000	1.4938
R_CB18	R_CB9	8.45	0.0000	1.4938
R_CB35	R_CB21	8.45	0.0000	1.4938
R_Caudal_boundary_Yaxis	R_CB10	8.45	0.0000	1.4938
R_CB4	R_CB35	8.44	0.0000	1.4920
R_Caudal_boundary_Yaxis	R_CB14	8.44	0.0000	1.4920
R_CB8	R_CB20	8.36	0.0000	1.4779
R_CB35	R_CB14	8.36	0.0000	1.4779
R_CB5	R_CB27	8.32	0.0000	1.4708
R_CB7	R_CB32	8.3	0.0000	1.4672
R_CB9	R_CB33	8.25	0.0000	1.4584
R_CB7	R_CB14	8.23	0.0000	1.4549
R_CB36	R_Dorsal_boundary_Zaxis	8.2	0.0000	1.4496
R_Caudal_boundary_Yaxis	R_CB26	8.2	0.0000	1.4496
R_CB31	R_Medial_boundary_Xaxis	8.2	0.0000	1.4496
R_CB39	R_CB18	8.2	0.0000	1.4496
R_CB7	R_Lateral_boundary_Xaxis	8.19	0.0000	1.4478
R_Caudal_boundary_Yaxis	R_CB24	8.19	0.0000	1.4478
R_CB8	R_CB27	8.19	0.0000	1.4478
R_CB30	R_CB31	8.19	0.0000	1.4478
R_Rostral_boundary_Yaxis	R_CB2	8.16	0.0000	1.4425
R_CB5	R_CB13	8.16	0.0000	1.4425
R_CB38	R_CB31	8.16	0.0000	1.4425

R_CB35	R_CB35	8.15	0.0000	1.4407
R_CB39	R_CB5	8.15	0.0000	1.4407
R_CB37	R_CB12	8.14	0.0000	1.4390
R_Rostral_boundary_Yaxis	R_CB25	8.12	0.0000	1.4354
R_CB39	R_CB21	8.12	0.0000	1.4354
R_Medial_boundary_Xaxis	R_CB23	8.09	0.0000	1.4301
R_CB9	R_Ventral_boundary_Zaxis	8.08	0.0000	1.4284
R_CB4	R_CB29	8.05	0.0000	1.4231
R_CB19	R_CB2	8.02	0.0000	1.4177
R_CB5	R_CB24	8.02	0.0000	1.4177
R_CB36	R_CB8	8	0.0000	1.4142
R_Dorsal_boundary_Zaxis	R_CB26	7.99	0.0000	1.4124
R_CB36	R_CB39	7.98	0.0000	1.4107
R_Dorsal_boundary_Zaxis	R_CB32	7.98	0.0000	1.4107
R_CB9	R_CB9	7.96	0.0000	1.4071
R_CB31	R_CB27	7.96	0.0000	1.4071
R_CB35	R_CB15	7.96	0.0000	1.4071
R_CB14	R_CB3	7.94	0.0000	1.4036
R_CB8	R_CB15	7.93	0.0000	1.4018
R_CB14	R_CB20	7.92	0.0000	1.4001
R_CB26	R_CB37	7.92	0.0000	1.4001
R_Dorsal_boundary_Zaxis	R_Caudal_boundary_Yaxis	7.92	0.0000	1.4001
R_CB18	R_CB9	7.87	0.0000	1.3912
R_Medial_boundary_Xaxis	R_CB8	7.87	0.0000	1.3912
R_CB5	R_CB39	7.86	0.0000	1.3895
R_CB35	R_CB36	7.86	0.0000	1.3895
R_Dorsal_boundary_Zaxis	R_CB32	7.85	0.0000	1.3877
R_CB8	R_CB10	7.84	0.0000	1.3859
R_CB9	R_CB4	7.83	0.0000	1.3842
R_CB36	R_CB8	7.81	0.0000	1.3806
R_Caudal_boundary_Yaxis	R_CB32	7.81	0.0000	1.3806
R_CB37	R_CB10	7.8	0.0000	1.3789
R_CB8	R_CB12	7.8	0.0000	1.3789
R_CB39	R_CB28	7.79	0.0000	1.3771
R_CB4	R_CB10	7.79	0.0000	1.3771
R_CB37	R_CB11	7.78	0.0000	1.3753
R_CB31	R_CB1	7.78	0.0000	1.3753
R_CB36	R_CB9	7.78	0.0000	1.3753
R_CB9	R_CB9	7.78	0.0000	1.3753
R_CB36	R_CB32	7.77	0.0000	1.3736
R_CB35	R_CB36	7.71	0.0000	1.3629
R_Caudal_boundary_Yaxis	R_CB31	7.71	0.0000	1.3629
R_CB14	R_CB30	7.65	0.0000	1.3523
R_CB5	R_CB21	7.65	0.0000	1.3523
R_CB18	R_CB12	7.6	0.0000	1.3435

R_CB38	R_CB14	7.6	0.0000	1.3435
R_CB26	R_CB18	7.58	0.0000	1.3400
R_CB26	R_CB18	7.58	0.0000	1.3400
R_CB14	R_CB12	7.58	0.0000	1.3400
R_CB9	R_CB9	7.55	0.0000	1.3347
R_CB4	R_CB19	7.55	0.0000	1.3347
R_CB9	R_CB9	7.54	0.0000	1.3329
R_CB31	R_CB14	7.54	0.0000	1.3329
R_CB18	R_CB18	7.52	0.0000	1.3294
R_Medial_boundary_Xaxis	R_CB17	7.49	0.0000	1.3241
R_CB15	R_CB32	7.49	0.0000	1.3241
R_CB14	R_CB5	7.42	0.0000	1.3117
R_Rostral_boundary_Yaxis	R_Dorsal_boundary_Zaxis	7.39	0.0000	1.3064
R_CB18	R_CB14	7.39	0.0000	1.3064
R_CB39	R_CB29	7.39	0.0000	1.3064
R_CB38	R_CB9	7.35	0.0000	1.2993
R_CB26	R_CB31	7.34	0.0000	1.2975
R_Caudal_boundary_Yaxis	R_CB4	7.34	0.0000	1.2975
R_Dorsal_boundary_Zaxis	R_CB8	7.28	0.0000	1.2869
R_CB35	R_CB2	7.28	0.0000	1.2869
R_CB8	R_CB18	7.28	0.0000	1.2869
R_Caudal_boundary_Yaxis	R_CB31	7.28	0.0000	1.2869
R_Dorsal_boundary_Zaxis	R_CB5	7.25	0.0000	1.2816
R_Rostral_boundary_Yaxis	R_CB9	7.25	0.0000	1.2816
R_CB36	R_CB22	7.25	0.0000	1.2816
R_CB4	R_Caudal_boundary_Yaxis	7.25	0.0000	1.2816
R_CB39	R_CB3	7.24	0.0000	1.2799
R_CB14	R_CB24	7.24	0.0000	1.2799
R_CB4	R_CB9	7.24	0.0000	1.2799
R_CB38	R_CB33	7.22	0.0000	1.2763
R_CB30	R_CB22	7.22	0.0000	1.2763
R_CB9	R_CB31	7.2	0.0000	1.2728
R_CB8	R_CB5	7.2	0.0000	1.2728
R_CB26	R_CB9	7.19	0.0000	1.2710
R_CB26	R_CB10	7.16	0.0000	1.2657
R_CB4	R_CB6	7.16	0.0000	1.2657
R_CB5	R_CB32	7.13	0.0000	1.2604
R_Dorsal_boundary_Zaxis	R_CB39	7.12	0.0000	1.2587
R_CB35	R_CB6	7.12	0.0000	1.2587
R_CB31	R_CB14	7.12	0.0000	1.2587
R_CB15	R_CB25	7.12	0.0000	1.2587
R_CB15	R_CB2	7.1	0.0000	1.2551
R_CB7	R_CB26	7.09	0.0000	1.2533
R_CB19	R_CB18	7.09	0.0000	1.2533
R_CB31	R_CB16	7.03	0.0000	1.2427

R_CB8	R_Rostral_boundary_Yaxis	7.01	0.0000	1.2392
R_CB8	R_CB15	7.01	0.0000	1.2392
R_Medial_boundary_Xaxis	R_Caudal_boundary_Yaxis	7	0.0000	1.2374
R_CB26	R_CB22	7	0.0000	1.2374
R_Medial_boundary_Xaxis	R_CB15	7	0.0000	1.2374
R_CB37	R_CB19	7	0.0000	1.2374
R_CB39	R_CB17	7	0.0000	1.2374
R_CB5	R_Caudal_boundary_Yaxis	6.98	0.0000	1.2339
R_Medial_boundary_Xaxis	R_CB32	6.98	0.0000	1.2339
R_CB36	R_CB18	6.96	0.0000	1.2304
R_CB15	R_CB31	6.96	0.0000	1.2304
R_CB18	R_CB4	6.96	0.0000	1.2304
R_CB37	R_CB27	6.95	0.0000	1.2286
R_CB7	R_CB32	6.95	0.0000	1.2286
R_CB9	R_CB26	6.95	0.0000	1.2286
R_CB14	R_CB2	6.94	0.0000	1.2268
R_Rostral_boundary_Yaxis	R_Caudal_boundary_Yaxis	6.9	0.0000	1.2198
R_CB36	R_Lateral_boundary_Xaxis	6.87	0.0000	1.2145
R_CB15	R_CB22	6.87	0.0000	1.2145
R_CB35	R_CB23	6.87	0.0000	1.2145
R_CB18	R_CB23	6.86	0.0000	1.2127
R_CB38	R_CB33	6.86	0.0000	1.2127
R_Caudal_boundary_Yaxis	R_CB2	6.86	0.0000	1.2127
R_CB19	R_Dorsal_boundary_Zaxis	6.83	0.0000	1.2074
R_CB15	R_Rostral_boundary_Yaxis	6.83	0.0000	1.2074
R_CB4	R_CB4	6.82	0.0000	1.2056
R_CB38	R_Dorsal_boundary_Zaxis	6.82	0.0000	1.2056
R_CB18	R_CB12	6.82	0.0000	1.2056
R_CB38	R_CB1	6.81	0.0000	1.2038
R_Caudal_boundary_Yaxis	R_CB8	6.81	0.0000	1.2038
R_CB4	R_CB8	6.81	0.0000	1.2038
R_CB36	R_CB11	6.81	0.0000	1.2038
R_Rostral_boundary_Yaxis	R_Caudal_boundary_Yaxis	6.79	0.0000	1.2003
R_Dorsal_boundary_Zaxis	R_CB39	6.79	0.0000	1.2003
R_CB30	R_CB9	6.79	0.0000	1.2003
R_Medial_boundary_Xaxis	R_CB30	6.77	0.0000	1.1968
R_CB8	R_CB14	6.75	0.0000	1.1932
R_CB39	R_CB36	6.75	0.0000	1.1932
R_Medial_boundary_Xaxis	R_Ventral_boundary_Zaxis	6.71	0.0000	1.1862
R_CB15	R_CB3	6.71	0.0000	1.1862
R_CB35	R_CB30	6.7	0.0000	1.1844
R_CB7	R_CB19	6.64	0.0000	1.1738
R_CB26	R_CB23	6.64	0.0000	1.1738
R_CB19	R_CB13	6.59	0.0000	1.1650
R_CB7	R_Medial_boundary_Xaxis	6.59	0.0000	1.1650

R_Ventral_boundary_Zaxis	R_CB7	6.58	0.0000	1.1632
R_CB36	R_CB24	6.57	0.0000	1.1614
R_CB5	R_Medial_boundary_Xaxis	6.57	0.0000	1.1614
R_Dorsal_boundary_Zaxis	R_CB32	6.57	0.0000	1.1614
R_CB9	R_CB8	6.55	0.0000	1.1579
R_CB36	R_CB26	6.53	0.0000	1.1544
R_CB37	R_CB33	6.53	0.0000	1.1544
R_CB37	R_CB4	6.53	0.0000	1.1544
R_CB5	R_CB20	6.53	0.0000	1.1544
R_Rostral_boundary_Yaxis	R_Medial_boundary_Xaxis	6.52	0.0000	1.1526
R_Medial_boundary_Xaxis	R_CB16	6.48	0.0000	1.1455
R_CB37	R_CB25	6.47	0.0000	1.1437
R_CB9	R_CB19	6.46	0.0000	1.1420
R_Rostral_boundary_Yaxis	R_CB35	6.46	0.0000	1.1420
R_CB19	R_Dorsal_boundary_Zaxis	6.45	0.0000	1.1402
R_CB19	R_CB5	6.44	0.0000	1.1384
R_CB36	R_CB5	6.44	0.0000	1.1384
R_CB37	R_CB33	6.44	0.0000	1.1384
R_CB31	R_CB37	6.44	0.0000	1.1384
R_Medial_boundary_Xaxis	R_CB24	6.44	0.0000	1.1384
R_CB38	R_CB15	6.43	0.0000	1.1367
R_CB37	R_Medial_boundary_Xaxis	6.43	0.0000	1.1367
R_CB14	R_CB15	6.42	0.0000	1.1349
R_Medial_boundary_Xaxis	R_CB6	6.42	0.0000	1.1349
R_CB7	R_CB33	6.41	0.0000	1.1331
R_CB7	R_CB12	6.41	0.0000	1.1331
R_CB14	R_Caudal_boundary_Yaxis	6.39	0.0000	1.1296
R_CB15	R_CB11	6.39	0.0000	1.1296
R_CB30	R_CB38	6.38	0.0000	1.1278
R_CB7	R_CB1	6.38	0.0000	1.1278
R_CB14	R_CB11	6.38	0.0000	1.1278
R_CB37	R_CB31	6.37	0.0000	1.1261
R_CB5	R_Caudal_boundary_Yaxis	6.37	0.0000	1.1261
R_CB30	R_CB22	6.37	0.0000	1.1261
R_CB38	R_CB5	6.35	0.0000	1.1225
R_Dorsal_boundary_Zaxis	R_CB16	6.35	0.0000	1.1225
R_CB38	R_CB35	6.31	0.0000	1.1155
R_CB31	R_CB20	6.31	0.0000	1.1155
R_CB8	R_CB37	6.3	0.0000	1.1137
R_CB38	R_CB13	6.29	0.0000	1.1119
R_CB15	R_CB26	6.29	0.0000	1.1119
R_CB26	R_Rostral_boundary_Yaxis	6.26	0.0000	1.1066
R_CB7	R_CB39	6.24	0.0000	1.1031
R_Rostral_boundary_Yaxis	R_CB2	6.23	0.0000	1.1013
R_CB14	R_CB9	6.22	0.0000	1.0996

R_CB30	R_Lateral_boundary_Xaxis	6.22	0.0000	1.0996
R_CB37	R_Medial_boundary_Xaxis	6.19	0.0000	1.0942
R_CB26	R_Ventral_boundary_Zaxis	6.19	0.0000	1.0942
R_CB39	R_CB7	6.16	0.0000	1.0889
R_Ventral_boundary_Zaxis	R_CB37	6.16	0.0000	1.0889
R_CB19	R_CB32	6.16	0.0000	1.0889
R_CB14	R_CB6	6.15	0.0000	1.0872
R_Dorsal_boundary_Zaxis	R_CB27	6.13	0.0000	1.0836
R_CB35	R_Medial_boundary_Xaxis	6.13	0.0000	1.0836
R_CB9	R_CB21	6.12	0.0000	1.0819
R_CB7	R_CB3	6.12	0.0000	1.0819
R_CB5	R_CB3	6.08	0.0000	1.0748
R_CB7	R_CB12	6.08	0.0000	1.0748
R_CB38	R_CB18	6.06	0.0000	1.0713
R_CB19	R_CB10	6.06	0.0000	1.0713
R_Medial_boundary_Xaxis	R_CB15	6.06	0.0000	1.0713
R_CB30	R_CB26	6.06	0.0000	1.0713
R_CB26	R_CB14	6.04	0.0000	1.0677
R_Ventral_boundary_Zaxis	R_Rostral_boundary_Yaxis	6.03	0.0000	1.0660
R_CB7	R_CB27	6.03	0.0000	1.0660
R_CB15	R_CB25	6	0.0000	1.0607
R_CB9	R_CB10	6	0.0000	1.0607
R_CB30	R_CB29	5.99	0.0000	1.0589
R_Caudal_boundary_Yaxis	R_CB10	5.97	0.0000	1.0554
R_Ventral_boundary_Zaxis	R_CB31	5.97	0.0000	1.0554
R_CB26	R_CB38	5.96	0.0000	1.0536
R_Rostral_boundary_Yaxis	R_CB36	5.94	0.0000	1.0501
R_CB15	R_CB28	5.94	0.0000	1.0501
R_CB8	R_Ventral_boundary_Zaxis	5.9	0.0000	1.0430
R_CB38	R_Dorsal_boundary_Zaxis	5.9	0.0000	1.0430
R_CB26	R_CB22	5.9	0.0000	1.0430
R_Rostral_boundary_Yaxis	R_CB20	5.89	0.0000	1.0412
R_Rostral_boundary_Yaxis	R_CB35	5.88	0.0000	1.0394
R_Rostral_boundary_Yaxis	R_CB36	5.88	0.0000	1.0394
R_CB39	R_CB15	5.88	0.0000	1.0394
R_CB18	R_CB18	5.88	0.0000	1.0394
R_Medial_boundary_Xaxis	R_CB11	5.87	0.0000	1.0377
R_CB35	R_CB11	5.87	0.0000	1.0377
R_CB36	R_CB35	5.87	0.0000	1.0377
R_Medial_boundary_Xaxis	R_CB32	5.87	0.0000	1.0377
R_Ventral_boundary_Zaxis	R_CB1	5.87	0.0000	1.0377
R_CB30	R_CB1	5.87	0.0000	1.0377
R_CB37	R_Ventral_boundary_Zaxis	5.86	0.0000	1.0359
R_Rostral_boundary_Yaxis	R_CB27	5.86	0.0000	1.0359
R_CB30	R_CB38	5.84	0.0000	1.0324

R_CB15	R_CB19	5.84	0.0000	1.0324
R_CB31	R_CB38	5.83	0.0000	1.0306
R_CB36	R_CB11	5.79	0.0000	1.0235
R_CB19	R_CB18	5.79	0.0000	1.0235
R_CB31	R_CB7	5.79	0.0000	1.0235
R_CB39	R_CB1	5.79	0.0000	1.0235
R_Ventral_boundary_Zaxis	R_CB21	5.79	0.0000	1.0235
R_CB5	R_CB5	5.78	0.0000	1.0218
R_CB31	R_Ventral_boundary_Zaxis	5.77	0.0000	1.0200
R_CB39	R_Rostral_boundary_Yaxis	5.74	0.0000	1.0147
R_CB37	R_Caudal_boundary_Yaxis	5.74	0.0000	1.0147
R_CB26	R_CB4	5.7	0.0000	1.0076
R_Ventral_boundary_Zaxis	R_CB18	5.7	0.0000	1.0076
R_CB4	R_CB38	5.69	0.0000	1.0059
R_CB35	R_CB38	5.67	0.0000	1.0023
R_CB15	R_Medial_boundary_Xaxis	5.66	0.0000	1.0006
R_CB30	R_CB30	5.66	0.0000	1.0006
R_CB18	R_CB36	5.64	0.0000	0.9970
R_Caudal_boundary_Yaxis	R_CB21	5.64	0.0000	0.9970
R_CB18	R_CB36	5.63	0.0000	0.9953
R_Ventral_boundary_Zaxis	R_CB16	5.63	0.0000	0.9953
R_CB4	R_CB8	5.62	0.0000	0.9935
R_CB18	R_CB39	5.6	0.0000	0.9899
R_Ventral_boundary_Zaxis	R_CB4	5.6	0.0000	0.9899
R_CB7	R_Medial_boundary_Xaxis	5.59	0.0000	0.9882
R_Ventral_boundary_Zaxis	R_CB4	5.58	0.0000	0.9864
R_CB18	R_CB22	5.58	0.0000	0.9864
R_CB19	R_CB30	5.57	0.0000	0.9846
R_Dorsal_boundary_Zaxis	R_CB39	5.55	0.0000	0.9811
R_Rostral_boundary_Yaxis	R_Dorsal_boundary_Zaxis	5.55	0.0000	0.9811
R_Ventral_boundary_Zaxis	R_CB28	5.55	0.0000	0.9811
R_Ventral_boundary_Zaxis	R_CB6	5.52	0.0000	0.9758
R_CB19	R_CB7	5.52	0.0000	0.9758
R_CB7	R_CB36	5.52	0.0000	0.9758
R_CB31	R_CB6	5.52	0.0000	0.9758
R_CB32	R_CB29	5.51	0.0000	0.9740
R_CB31	R_CB14	5.49	0.0000	0.9705
R_CB27	R_CB33	5.49	0.0000	0.9705
R_CB26	R_CB26	5.49	0.0000	0.9705
R_CB15	R_CB19	5.48	0.0000	0.9687
R_CB32	R_CB8	5.48	0.0000	0.9687
R_CB9	R_CB33	5.45	0.0000	0.9634
R_CB15	R_CB25	5.44	0.0000	0.9617
R_CB33	R_CB31	5.44	0.0000	0.9617
R_CB9	R_CB6	5.44	0.0000	0.9617

R_CB33	R_CB37	5.43	0.0000	0.9599
R_CB31	R_CB7	5.43	0.0000	0.9599
R_CB6	R_CB15	5.42	0.0000	0.9581
R_CB9	R_Caudal_boundary_Yaxis	5.42	0.0000	0.9581
R_CB6	R_CB24	5.42	0.0000	0.9581
R_CB5	R_CB14	5.4	0.0000	0.9546
R_CB28	R_CB32	5.4	0.0000	0.9546
R_CB20	R_Caudal_boundary_Yaxis	5.38	0.0000	0.9511
R_CB32	R_CB3	5.38	0.0000	0.9511
R_CB26	R_CB39	5.37	0.0000	0.9493
R_CB27	R_CB29	5.37	0.0000	0.9493
R_CB23	R_CB4	5.36	0.0000	0.9475
R_CB26	R_CB6	5.36	0.0000	0.9475
R_CB9	R_CB18	5.35	0.0000	0.9458
R_CB15	R_CB8	5.35	0.0000	0.9458
R_CB15	R_CB6	5.35	0.0000	0.9458
R_CB32	R_CB20	5.34	0.0000	0.9440
R_Dorsal_boundary_Zaxis	R_CB36	5.33	0.0000	0.9422
R_CB19	R_CB21	5.33	0.0000	0.9422
R_CB17	R_Ventral_boundary_Zaxis	5.33	0.0000	0.9422
R_Ventral_boundary_Zaxis	R_CB36	5.3	0.0000	0.9369
R_CB24	R_CB27	5.29	0.0000	0.9351
R_CB26	R_CB37	5.29	0.0000	0.9351
R_CB20	R_CB19	5.27	0.0000	0.9316
R_Dorsal_boundary_Zaxis	R_CB5	5.24	0.0000	0.9263
R_CB9	R_CB30	5.24	0.0000	0.9263
R_CB31	R_Rostral_boundary_Yaxis	5.21	0.0000	0.9210
R_Ventral_boundary_Zaxis	R_CB26	5.18	0.0000	0.9157
R_CB32	R_CB36	5.18	0.0000	0.9157
R_CB30	R_Lateral_boundary_Xaxis	5.16	0.0000	0.9122
R_CB29	R_Dorsal_boundary_Zaxis	5.15	0.0000	0.9104
R_CB9	R_CB33	5.15	0.0000	0.9104
R_CB21	R_CB37	5.15	0.0000	0.9104
R_CB27	R_CB37	5.14	0.0000	0.9086
R_Caudal_boundary_Yaxis	R_CB31	5.12	0.0000	0.9051
R_CB24	R_CB12	5.12	0.0000	0.9051
R_Ventral_boundary_Zaxis	R_Rostral_boundary_Yaxis	5.08	0.0000	0.8980
R_CB2	R_CB5	5.08	0.0000	0.8980
R_CB5	R_CB19	5.07	0.0000	0.8963
R_CB17	R_CB10	5.07	0.0000	0.8963
R_CB15	R_CB39	5.06	0.0000	0.8945
R_CB6	R_Lateral_boundary_Xaxis	5.06	0.0000	0.8945
R_Caudal_boundary_Yaxis	R_Rostral_boundary_Yaxis	5.05	0.0000	0.8927
R_CB32	R_CB17	5.05	0.0000	0.8927
R_CB5	R_CB31	5.05	0.0000	0.8927

R_Ventral_boundary_Zaxis	R_CB15	5.04	0.0000	0.8910
R_CB5	R_CB29	5.04	0.0000	0.8910
R_CB2	R_CB1	5.02	0.0000	0.8874
R_Caudal_boundary_Yaxis	R_CB27	5.02	0.0000	0.8874
R_Dorsal_boundary_Zaxis	R_CB29	5	0.0000	0.8839
R_CB12	R_CB21	4.98	0.0000	0.8803
R_CB9	R_CB38	4.97	0.0000	0.8786
R_CB33	R_CB27	4.96	0.0000	0.8768
R_CB22	R_CB19	4.95	0.0000	0.8750
R_Ventral_boundary_Zaxis	R_CB5	4.95	0.0000	0.8750
R_CB5	R_CB19	4.95	0.0000	0.8750
R_CB29	R_Medial_boundary_Xaxis	4.95	0.0000	0.8750
R_CB25	R_CB3	4.93	0.0000	0.8715
R_Ventral_boundary_Zaxis	R_CB39	4.9	0.0000	0.8662
R_CB28	R_CB17	4.9	0.0000	0.8662
R_CB30	R_CB9	4.9	0.0000	0.8662
R_Medial_boundary_Xaxis	R_CB13	4.89	0.0000	0.8644
R_CB31	R_CB38	4.89	0.0000	0.8644
R_CB28	R_CB33	4.89	0.0000	0.8644
R_CB9	R_CB36	4.89	0.0000	0.8644
R_CB33	R_Medial_boundary_Xaxis	4.87	0.0000	0.8609
R_CB35	R_CB2	4.86	0.0000	0.8591
R_CB21	R_CB8	4.86	0.0000	0.8591
R_CB19	R_CB14	4.85	0.0000	0.8574
R_CB20	R_CB6	4.82	0.0000	0.8521
R_CB29	R_Medial_boundary_Xaxis	4.82	0.0000	0.8521
R_CB5	R_CB30	4.81	0.0000	0.8503
R_CB33	R_Medial_boundary_Xaxis	4.8	0.0000	0.8485
R_CB39	R_CB31	4.8	0.0000	0.8485
R_CB24	R_CB13	4.8	0.0000	0.8485
R_CB6	R_CB36	4.8	0.0000	0.8485
R_CB31	R_CB18	4.79	0.0000	0.8468
R_CB12	R_CB37	4.78	0.0000	0.8450
R_CB28	R_CB30	4.78	0.0000	0.8450
R_Ventral_boundary_Zaxis	R_CB37	4.78	0.0000	0.8450
R_CB26	R_CB1	4.78	0.0000	0.8450
R_CB39	R_CB39	4.78	0.0000	0.8450
R_CB24	R_CB37	4.78	0.0000	0.8450
R_CB15	R_CB37	4.78	0.0000	0.8450
R_CB9	R_CB38	4.77	0.0000	0.8432
R_CB2	R_CB30	4.76	0.0000	0.8415
R_CB32	R_CB33	4.76	0.0000	0.8415
R_CB32	R_CB20	4.76	0.0000	0.8415
R_CB32	R_Lateral_boundary_Xaxis	4.75	0.0000	0.8397
R_CB22	R_CB31	4.75	0.0000	0.8397

R_CB21	R_CB1	4.75	0.0000	0.8397
R_CB16	R_CB24	4.74	0.0000	0.8379
R_CB12	R_Dorsal_boundary_Zaxis	4.74	0.0000	0.8379
R_CB15	R_CB33	4.73	0.0000	0.8362
R_CB12	R_CB7	4.73	0.0000	0.8362
R_CB6	R_CB31	4.73	0.0000	0.8362
R_CB16	R_CB35	4.72	0.0000	0.8344
R_CB12	R_CB17	4.72	0.0000	0.8344
R_CB8	R_Rostral_boundary_Yaxis	4.72	0.0000	0.8344
R_CB14	R_Medial_boundary_Xaxis	4.72	0.0000	0.8344
R_CB18	R_CB14	4.72	0.0000	0.8344
R_CB16	R_CB37	4.65	0.0000	0.8220
R_CB31	R_CB28	4.59	0.0000	0.8114
R_Dorsal_boundary_Zaxis	R_Medial_boundary_Xaxis	4.59	0.0000	0.8114
R_CB28	R_CB37	4.58	0.0000	0.8096
R_CB28	R_CB13	4.57	0.0000	0.8079
R_CB22	R_CB32	4.57	0.0000	0.8079
R_CB9	R_CB38	4.57	0.0000	0.8079
R_CB31	R_CB39	4.57	0.0000	0.8079
R_CB2	R_CB38	4.57	0.0000	0.8079
R_CB21	R_CB25	4.57	0.0000	0.8079
R_Ventral_boundary_Zaxis	R_CB13	4.57	0.0000	0.8079
R_CB16	R_CB9	4.57	0.0000	0.8079
R_CB28	R_CB35	4.56	0.0000	0.8061
R_CB4	R_Medial_boundary_Xaxis	4.54	0.0000	0.8026
R_CB22	R_CB16	4.54	0.0000	0.8026
R_CB32	R_CB8	4.5	0.0000	0.7955
R_CB17	R_CB21	4.5	0.0000	0.7955
R_CB26	R_CB10	4.47	0.0000	0.7902
R_CB15	R_CB5	4.47	0.0000	0.7902
R_CB15	R_CB38	4.46	0.0000	0.7884
R_CB29	R_Rostral_boundary_Yaxis	4.46	0.0000	0.7884
R_Caudal_boundary_Yaxis	R_CB36	4.45	0.0000	0.7867
R_CB4	R_CB22	4.45	0.0000	0.7867
R_CB5	R_CB33	4.45	0.0000	0.7867
R_Caudal_boundary_Yaxis	R_Medial_boundary_Xaxis	4.45	0.0000	0.7867
R_CB12	R_CB37	4.45	0.0000	0.7867
R_CB33	R_CB33	4.45	0.0000	0.7867
R_CB9	R_Dorsal_boundary_Zaxis	4.45	0.0000	0.7867
R_CB16	R_CB33	4.44	0.0000	0.7849
R_CB19	R_Dorsal_boundary_Zaxis	4.43	0.0000	0.7831
R_CB23	R_CB8	4.39	0.0000	0.7760
R_CB23	R_Lateral_boundary_Xaxis	4.38	0.0000	0.7743
R_CB32	R_Caudal_boundary_Yaxis	4.37	0.0000	0.7725
R_CB13	R_CB14	4.37	0.0000	0.7725

R_CB9	R_CB30	4.37	0.0000	0.7725
R_CB24	R_CB27	4.37	0.0000	0.7725
R_Ventral_boundary_Zaxis	R_CB7	4.37	0.0000	0.7725
R_CB23	R_CB35	4.37	0.0000	0.7725
R_CB20	R_CB38	4.37	0.0000	0.7725
R_CB24	R_Ventral_boundary_Zaxis	4.36	0.0000	0.7707
R_CB19	R_CB30	4.36	0.0000	0.7707
R_CB6	R_CB8	4.36	0.0000	0.7707
R_CB5	R_CB37	4.34	0.0000	0.7672
R_CB22	R_CB13	4.34	0.0000	0.7672
R_CB5	R_CB2	4.33	0.0000	0.7654
R_CB20	R_CB19	4.33	0.0000	0.7654
R_Medial_boundary_Xaxis	R_CB17	4.33	0.0000	0.7654
R_CB13	R_CB26	4.32	0.0000	0.7637
R_Medial_boundary_Xaxis	R_CB31	4.32	0.0000	0.7637
R_CB26	R_CB38	4.32	0.0000	0.7637
R_CB35	R_CB39	4.32	0.0000	0.7637
R_CB6	R_Ventral_boundary_Zaxis	4.32	0.0000	0.7637
R_CB2	R_CB15	4.31	0.0000	0.7619
R_CB2	R_CB33	4.31	0.0000	0.7619
R_CB36	R_CB18	4.31	0.0000	0.7619
R_CB27	R_CB22	4.31	0.0000	0.7619
R_CB26	R_CB37	4.28	0.0000	0.7566
R_CB26	R_CB1	4.27	0.0000	0.7548
R_CB6	R_CB23	4.27	0.0000	0.7548
R_CB7	R_CB29	4.27	0.0000	0.7548
R_CB36	R_CB11	4.27	0.0000	0.7548
R_CB27	R_CB29	4.27	0.0000	0.7548
R_CB20	R_CB14	4.25	0.0000	0.7513
R_CB38	R_CB26	4.25	0.0000	0.7513
R_CB5	R_CB36	4.25	0.0000	0.7513
R_Rostral_boundary_Yaxis	R_CB17	4.25	0.0000	0.7513
R_CB21	R_CB8	4.24	0.0000	0.7495
R_Ventral_boundary_Zaxis	R_CB27	4.24	0.0000	0.7495
R_CB18	R_CB25	4.23	0.0000	0.7478
R_CB16	R_Caudal_boundary_Yaxis	4.23	0.0000	0.7478
R_Ventral_boundary_Zaxis	R_CB19	4.22	0.0000	0.7460
R_CB21	R_Medial_boundary_Xaxis	4.21	0.0000	0.7442
R_CB27	R_CB24	4.21	0.0000	0.7442
R_CB10	R_CB3	4.2	0.0001	0.7425
R_CB4	R_CB20	4.2	0.0001	0.7425
R_CB6	R_CB26	4.2	0.0000	0.7425
R_CB8	R_CB2	4.2	0.0000	0.7425
R_CB32	R_CB31	4.17	0.0001	0.7372
R_CB33	R_CB38	4.17	0.0001	0.7372

R_CB17	R_CB2	4.17	0.0001	0.7372
R_Caudal_boundary_Yaxis	R_Dorsal_boundary_Zaxis	4.16	0.0001	0.7354
R_CB39	R_CB7	4.16	0.0001	0.7354
R_CB4	R_CB16	4.14	0.0001	0.7319
R_CB30	R_CB12	4.13	0.0001	0.7301
R_Caudal_boundary_Yaxis	R_CB21	4.13	0.0001	0.7301
R_CB29	R_CB36	4.13	0.0001	0.7301
R_CB25	R_CB35	4.1	0.0001	0.7248
R_CB12	R_CB7	4.1	0.0001	0.7248
R_CB20	R_CB26	4.09	0.0001	0.7230
R_CB20	R_CB20	4.09	0.0001	0.7230
R_CB20	R_CB21	4.08	0.0001	0.7212
R_CB27	R_CB14	4.08	0.0001	0.7212
R_CB6	R_CB19	4.02	0.0001	0.7106
R_CB8	R_CB13	4.02	0.0001	0.7106
R_CB8	R_CB5	4.02	0.0001	0.7106
R_CB29	R_Rostral_boundary_Yaxis	4.02	0.0001	0.7106
R_CB38	R_CB36	4	0.0001	0.7071
R_CB14	R_CB2	3.99	0.0001	0.7053
R_CB38	R_CB5	3.99	0.0001	0.7053
R_CB3	R_CB13	3.99	0.0001	0.7053
R_CB18	R_CB37	3.99	0.0001	0.7053
R_CB33	R_CB30	3.99	0.0001	0.7053
R_CB12	R_Lateral_boundary_Xaxis	3.98	0.0001	0.7036
R_CB31	R_Medial_boundary_Xaxis	3.96	0.0001	0.7000
R_CB15	R_CB16	3.96	0.0001	0.7000
R_CB19	R_CB9	3.96	0.0001	0.7000
R_CB30	R_CB8	3.96	0.0001	0.7000
R_Lateral_boundary_Xaxis	R_Medial_boundary_Xaxis	3.93	0.0001	0.6947
R_CB16	R_CB4	3.93	0.0001	0.6947
R_CB29	R_CB38	3.93	0.0001	0.6947
R_CB31	R_Medial_boundary_Xaxis	3.93	0.0001	0.6947
R_CB16	R_CB18	3.92	0.0001	0.6930
R_CB10	R_CB33	3.92	0.0001	0.6930
R_CB7	R_CB32	3.91	0.0002	0.6912
R_CB38	R_CB38	3.91	0.0002	0.6912
R_CB17	R_CB19	3.91	0.0002	0.6912
R_CB21	R_Ventral_boundary_Zaxis	3.91	0.0002	0.6912
R_CB39	R_CB28	3.9	0.0002	0.6894
R_CB38	R_CB33	3.9	0.0002	0.6894
R_CB36	R_CB19	3.9	0.0002	0.6894
R_CB10	R_CB11	3.9	0.0002	0.6894
R_CB35	R_CB1	3.9	0.0002	0.6894
R_CB33	R_CB33	3.9	0.0002	0.6894
R_CB13	R_CB4	3.89	0.0002	0.6877

R_CB17	R_CB37	3.87	0.0002	0.6841
R_CB7	R_CB10	3.86	0.0002	0.6824
R_CB31	R_CB10	3.86	0.0002	0.6824
R_CB31	R_CB11	3.86	0.0002	0.6824
R_CB20	R_CB7	3.84	0.0002	0.6788
R_CB13	R_CB11	3.83	0.0002	0.6771
R_Caudal_boundary_Yaxis	R_CB20	3.83	0.0002	0.6771
R_CB28	R_CB28	3.83	0.0002	0.6771
R_CB38	R_CB1	3.83	0.0002	0.6771
R_Medial_boundary_Xaxis	R_Medial_boundary_Xaxis	3.83	0.0002	0.6771
R_Caudal_boundary_Yaxis	R_CB18	3.81	0.0002	0.6735
R_CB23	R_CB14	3.81	0.0002	0.6735
R_CB32	R_Lateral_boundary_Xaxis	3.81	0.0002	0.6735
R_Dorsal_boundary_Zaxis	R_CB10	3.81	0.0002	0.6735
R_CB30	R_CB35	3.81	0.0002	0.6735
R_CB9	R_CB38	3.81	0.0002	0.6735
R_CB37	R_CB9	3.8	0.0002	0.6718
R_CB12	R_CB3	3.8	0.0002	0.6718
R_CB38	R_CB38	3.8	0.0002	0.6718
R_CB38	R_CB30	3.8	0.0002	0.6718
R_CB13	R_CB17	3.8	0.0002	0.6718
R_CB35	R_CB25	3.8	0.0002	0.6718
R_Medial_boundary_Xaxis	R_CB25	3.8	0.0002	0.6718
R_CB21	R_CB31	3.8	0.0002	0.6718
R_Medial_boundary_Xaxis	R_CB26	3.8	0.0002	0.6718
R_CB33	R_CB15	3.79	0.0002	0.6700
R_CB33	R_Ventral_boundary_Zaxis	3.79	0.0002	0.6700
R_CB8	R_Rostral_boundary_Yaxis	3.79	0.0002	0.6700
R_CB36	R_Caudal_boundary_Yaxis	3.79	0.0002	0.6700
R_CB29	R_Medial_boundary_Xaxis	3.77	0.0002	0.6664
R_CB22	R_CB21	3.77	0.0002	0.6664
R_CB37	R_CB1	3.76	0.0003	0.6647
R_Medial_boundary_Xaxis	R_CB18	3.74	0.0003	0.6611
R_CB33	R_CB37	3.74	0.0003	0.6611
R_CB13	R_CB39	3.7	0.0003	0.6541
R_CB10	R_CB19	3.7	0.0003	0.6541
R_CB4	R_CB20	3.7	0.0003	0.6541
R_CB37	R_CB39	3.7	0.0003	0.6541
R_Lateral_boundary_Xaxis	R_Medial_boundary_Xaxis	3.69	0.0003	0.6523
R_CB15	R_CB19	3.69	0.0003	0.6523
R_CB36	R_Lateral_boundary_Xaxis	3.69	0.0003	0.6523
R_CB23	R_CB23	3.69	0.0003	0.6523
R_CB8	R_CB30	3.68	0.0003	0.6505
R_Caudal_boundary_Yaxis	R_CB14	3.68	0.0003	0.6505
R_CB31	R_Caudal_boundary_Yaxis	3.68	0.0003	0.6505

R_CB2	R_CB2	3.68	0.0003	0.6505
R_CB19	R_Lateral_boundary_Xaxis	3.67	0.0004	0.6488
R_CB17	R_CB19	3.66	0.0004	0.6470
R_CB24	R_CB22	3.66	0.0004	0.6470
R_CB2	R_CB15	3.65	0.0004	0.6452
R_CB36	R_CB18	3.65	0.0004	0.6452
R_Medial_boundary_Xaxis	R_CB23	3.65	0.0004	0.6452
R_CB16	R_CB37	3.64	0.0004	0.6435
R_CB38	R_CB24	3.63	0.0004	0.6417
R_CB12	R_CB18	3.63	0.0004	0.6417
R_CB21	R_CB19	3.62	0.0004	0.6399
R_CB20	R_CB23	3.62	0.0004	0.6399
R_CB21	R_Lateral_boundary_Xaxis	3.62	0.0004	0.6399
R_CB5	R_Rostral_boundary_Yaxis	3.61	0.0004	0.6382
R_CB14	R_CB9	3.61	0.0004	0.6382
R_CB38	R_CB35	3.6	0.0004	0.6364
R_Rostral_boundary_Yaxis	R_CB36	3.58	0.0005	0.6329
R_CB28	R_Lateral_boundary_Xaxis	3.58	0.0005	0.6329
R_CB27	R_Rostral_boundary_Yaxis	3.58	0.0005	0.6329
R_CB27	R_CB8	3.58	0.0005	0.6329
R_CB20	R_Rostral_boundary_Yaxis	3.57	0.0005	0.6311
R_Rostral_boundary_Yaxis	R_CB2	3.57	0.0005	0.6311
R_CB18	R_CB35	3.54	0.0006	0.6258
R_CB10	R_Rostral_boundary_Yaxis	3.53	0.0006	0.6240
R_CB35	R_CB35	3.53	0.0006	0.6240
R_CB25	R_CB38	3.52	0.0006	0.6223
R_CB25	R_CB26	3.52	0.0006	0.6223
R_Lateral_boundary_Xaxis	R_CB10	3.51	0.0006	0.6205
R_CB37	R_CB30	3.51	0.0006	0.6205
R_CB14	R_CB10	3.5	0.0006	0.6187
R_CB23	R_CB36	3.5	0.0006	0.6187
R_CB2	R_CB37	3.5	0.0006	0.6187
R_CB39	R_CB10	3.5	0.0006	0.6187
R_CB22	R_Caudal_boundary_Yaxis	3.5	0.0006	0.6187
R_CB3	R_CB39	3.48	0.0007	0.6152
R_Medial_boundary_Xaxis	R_CB1	3.48	0.0007	0.6152
R_CB23	R_CB11	3.48	0.0007	0.6152
R_CB18	R_CB39	3.48	0.0007	0.6152
R_CB37	R_CB38	3.47	0.0007	0.6134
R_CB37	R_Lateral_boundary_Xaxis	3.47	0.0007	0.6134
R_CB19	R_CB22	3.45	0.0008	0.6099
R_CB17	R_CB10	3.44	0.0008	0.6081
R_CB19	R_CB15	3.44	0.0008	0.6081
R_CB39	R_CB36	3.43	0.0008	0.6063
R_Lateral_boundary_Xaxis	R_CB24	3.43	0.0008	0.6063

R_CB18	R_CB18	3.42	0.0008	0.6046
R_CB36	R_CB39	3.42	0.0008	0.6046
R_Lateral_boundary_Xaxis	R_CB7	3.4	0.0009	0.6010
R_CB35	R_CB27	3.4	0.0009	0.6010
R_Caudal_boundary_Yaxis	R_CB11	3.39	0.0009	0.5993
R_CB37	R_CB1	3.39	0.0009	0.5993
R_CB27	R_CB30	3.39	0.0009	0.5993
R_CB10	R_CB1	3.39	0.0009	0.5993
R_CB22	R_CB7	3.38	0.0009	0.5975
R_CB32	R_Lateral_boundary_Xaxis	3.38	0.0010	0.5975
R_CB39	R_CB32	3.38	0.0009	0.5975
R_CB10	R_CB14	3.37	0.0010	0.5957
R_Lateral_boundary_Xaxis	R_CB28	3.37	0.0010	0.5957
R_CB28	R_CB33	3.36	0.0010	0.5940
R_CB33	R_Rostral_boundary_Yaxis	3.36	0.0010	0.5940
R_CB39	R_CB29	3.34	0.0011	0.5904
R_CB30	R_Ventral_boundary_Zaxis	3.34	0.0011	0.5904
R_CB8	R_CB12	3.32	0.0012	0.5869
R_CB7	R_CB4	3.32	0.0012	0.5869
R_CB35	R_CB22	3.31	0.0012	0.5851
R_Caudal_boundary_Yaxis	R_CB8	3.31	0.0012	0.5851
R_CB37	R_CB20	3.3	0.0013	0.5834
R_CB29	R_CB20	3.3	0.0013	0.5834
R_Lateral_boundary_Xaxis	R_CB11	3.3	0.0013	0.5834
R_CB4	R_Lateral_boundary_Xaxis	3.29	0.0013	0.5816
R_CB21	R_CB38	3.29	0.0013	0.5816
R_Rostral_boundary_Yaxis	R_CB22	3.29	0.0013	0.5816
R_CB39	R_CB18	3.29	0.0013	0.5816
R_CB24	R_CB35	3.27	0.0014	0.5781
R_CB13	R_CB12	3.26	0.0014	0.5763
R_CB39	R_CB37	3.26	0.0014	0.5763
R_Caudal_boundary_Yaxis	R_CB21	3.25	0.0015	0.5745
R_CB13	R_CB24	3.24	0.0015	0.5728
R_CB39	R_CB30	3.23	0.0016	0.5710
R_CB35	R_CB13	3.22	0.0016	0.5692
R_CB35	R_Medial_boundary_Xaxis	3.22	0.0016	0.5692
R_CB24	R_CB39	3.22	0.0016	0.5692
R_CB8	R_Rostral_boundary_Yaxis	3.22	0.0016	0.5692
R_CB24	R_CB29	3.21	0.0017	0.5675
R_CB23	R_CB37	3.21	0.0017	0.5675
R_CB8	R_CB24	3.21	0.0017	0.5675
R_Lateral_boundary_Xaxis	R_CB1	3.2	0.0017	0.5657
R_Lateral_boundary_Xaxis	R_CB21	3.2	0.0017	0.5657
R_Dorsal_boundary_Zaxis	R_CB11	3.2	0.0017	0.5657
R_CB10	R_CB11	3.17	0.0019	0.5604

R_CB2	R_CB13	3.17	0.0019	0.5604
R_CB18	R_CB1	3.17	0.0019	0.5604
R_Medial_boundary_Xaxis	R_CB13	3.17	0.0019	0.5604
R_CB32	R_CB39	3.16	0.0020	0.5586
R_CB7	R_CB23	3.16	0.0020	0.5586
R_CB6	R_CB30	3.15	0.0020	0.5568
R_CB35	R_Caudal_boundary_Yaxis	3.15	0.0020	0.5568
R_CB22	R_CB13	3.15	0.0020	0.5568
R_CB23	R_Caudal_boundary_Yaxis	3.15	0.0020	0.5568
R_CB24	R_CB8	3.14	0.0021	0.5551
R_CB14	R_Dorsal_boundary_Zaxis	3.14	0.0021	0.5551
R_Ventral_boundary_Zaxis	R_CB28	3.14	0.0021	0.5551
R_CB4	R_CB25	3.14	0.0021	0.5551
R_CB37	R_CB24	3.13	0.0022	0.5533
R_CB15	R_CB25	3.12	0.0022	0.5515
R_CB25	R_CB23	3.11	0.0023	0.5498
R_CB5	R_CB35	3.11	0.0023	0.5498
R_CB7	R_CB37	3.1	0.0023	0.5480
R_Lateral_boundary_Xaxis	R_CB17	3.1	0.0023	0.5480
R_CB13	R_CB8	3.05	0.0028	0.5392
R_CB14	R_CB24	3.05	0.0028	0.5392
R_Medial_boundary_Xaxis	R_Rostral_boundary_Yaxis	3.04	0.0028	0.5374
R_Caudal_boundary_Yaxis	R_Lateral_boundary_Xaxis	3.04	0.0029	0.5374
R_CB3	R_CB30	3.04	0.0029	0.5374
R_CB20	R_CB6	3.04	0.0029	0.5374
R_CB39	R_CB26	3.04	0.0029	0.5374
R_Lateral_boundary_Xaxis	R_CB24	3.04	0.0028	0.5374
R_CB4	R_Dorsal_boundary_Zaxis	3.02	0.0031	0.5339
R_CB10	R_Lateral_boundary_Xaxis	3.02	0.0031	0.5339
R_CB6	R_CB12	2.98	0.0035	0.5268
R_CB14	R_CB19	2.98	0.0034	0.5268
R_CB3	R_CB31	2.98	0.0034	0.5268
R_CB25	R_CB11	2.98	0.0035	0.5268
Hipp_L_Ant	R_CB1	2.98	0.0035	0.5268
Hipp_L_Ant_3_Rev2	R_CB36	2.95	0.0038	0.5215
Hipp_L_Ant_2	R_CB3	2.95	0.0038	0.5215
Hipp_L_Ant_1	R_CB11	2.93	0.0041	0.5180
Hipp_L_Ant_3_Rev2	R_CB1	2.93	0.0041	0.5180
Hipp_L_Ant_1	R_CB25	2.93	0.0041	0.5180
Hipp_L_Ant_2	R_CB10	2.93	0.0041	0.5180
Hipp_L_Ant	R_Ventral_boundary_Zaxis	2.91	0.0043	0.5144
Hipp_L_Post2	R_CB1	2.9	0.0044	0.5127
Hipp_L_Post1	R_CB11	2.9	0.0044	0.5127
Hipp_L_Post2	R_CB29	2.84	0.0052	0.5020
Hipp_L_Post3	R_CB10	2.84	0.0053	0.5020

Hipp_L_Ant_1	R_CB7	2.84	0.0053	0.5020
Hipp_L_Ant	R_CB15	2.83	0.0054	0.5003
Hipp_L_Ant_1	R_CB2	2.83	0.0055	0.5003
Hipp_L_Post3	R_CB21	2.81	0.0058	0.4967
Hipp_L_Ant_1	R_CB26	2.77	0.0064	0.4897
Hipp_L_Post2	R_CB29	2.76	0.0066	0.4879
Hipp_L_Ant_3_Rev2	R_CB39	2.74	0.0069	0.4844
Hipp_L_Ant_2	R_CB5	2.74	0.0069	0.4844
Hipp_L_Post3	R_CB4	2.74	0.0070	0.4844
Hipp_L_Post1	R_CB19	2.74	0.0070	0.4844
Hipp_L_Ant_3_Rev2	R_CB18	2.7	0.0079	0.4773
Hipp_L_Post3	R_CB31	2.7	0.0079	0.4773
Hipp_L_Ant_2	R_Rostral_boundary_Yaxis	2.68	0.0084	0.4738
Hipp_L_Post2	R_CB6	2.68	0.0084	0.4738
Hipp_L_Post3	R_CB26	2.68	0.0082	0.4738
Hipp_L_Post3	R_CB30	2.62	0.0099	0.4632
Hipp_L_Ant_2	R_CB35	2.62	0.0099	0.4632
Hipp_L_Ant	R_CB11	2.62	0.0098	0.4632
Hipp_L_Ant_2	R_CB1	2.62	0.0098	0.4632
Hipp_L_Post1	R_Dorsal_boundary_Zaxis	2.62	0.0098	0.4632
Hipp_L_Post2	R_Dorsal_boundary_Zaxis	2.62	0.0098	0.4632
Hipp_L_Ant	R_CB15	2.6	0.0103	0.4596
Hipp_L_Post2	R_CB4	2.6	0.0103	0.4596
Hipp_L_Ant_3_Rev2	R_CB14	2.57	0.0114	0.4543
Hipp_L_Post1	R_Ventral_boundary_Zaxis	2.57	0.0113	0.4543
Hipp_L_Ant_1	R_CB3	2.56	0.0116	0.4525
R_CB11	R_CB35	2.56	0.0117	0.4525
R_CB1	R_CB22	2.56	0.0115	0.4525
R_CB1	R_CB14	2.56	0.0115	0.4525
R_CB11	R_CB13	2.56	0.0117	0.4525
R_CB36	R_CB4	2.52	0.0131	0.4455
R_CB1	R_CB2	2.51	0.0135	0.4437
R_CB11	R_CB22	2.49	0.0142	0.4402
R_CB9	R_CB18	2.49	0.0140	0.4402
R_CB11	R_CB37	2.49	0.0142	0.4402
R_CB1	R_CB25	2.49	0.0140	0.4402
R_CB11	R_CB7	2.46	0.0151	0.4349
R_CB1	R_CB8	2.46	0.0151	0.4349
R_CB1	R_CB24	2.45	0.0158	0.4331
R_CB11	R_CB25	2.45	0.0155	0.4331
R_CB1	R_CB14	2.45	0.0158	0.4331
R_CB35	R_CB2	2.44	0.0162	0.4313
R_CB11	R_CB4	2.41	0.0175	0.4260
R_CB1	R_CB18	2.39	0.0181	0.4225
R_CB11	R_CB26	2.39	0.0181	0.4225

R_CB1	R_CB37	2.39	0.0181	0.4225
R_CB7	R_Caudal_boundary_Yaxis	2.32	0.0219	0.4101
R_CB1	R_CB30	2.32	0.0217	0.4101
R_CB11	R_CB12	2.32	0.0217	0.4101
R_CB11	R_CB18	2.32	0.0219	0.4101
R_CB1	R_CB13	2.32	0.0220	0.4101
R_CB39	R_CB8	2.32	0.0220	0.4101
R_CB11	R_CB15	2.29	0.0234	0.4048
R_CB30	R_Ventral_boundary_Zaxis	2.27	0.0249	0.4013
R_CB11	R_CB11	2.26	0.0256	0.3995
R_Dorsal_boundary_Zaxis	R_Lateral_boundary_Xaxis	2.26	0.0256	0.3995
R_CB1	R_Ventral_boundary_Zaxis	2.26	0.0258	0.3995
R_CB26	R_CB1	2.26	0.0256	0.3995
R_CB1	R_CB28	2.24	0.0266	0.3960
R_CB2	R_Rostral_boundary_Yaxis	2.22	0.0281	0.3924
R_CB1	R_CB18	2.22	0.0281	0.3924
R_CB11	R_CB17	2.21	0.0287	0.3907
R_CB11	R_CB2	2.21	0.0292	0.3907
R_CB1	R_CB10	2.21	0.0290	0.3907
R_CB6	R_CB39	2.21	0.0290	0.3907
R_CB1	R_CB14	2.2	0.0292	0.3889
R_CB27	R_CB19	2.18	0.0312	0.3854
R_CB1	R_Ventral_boundary_Zaxis	2.18	0.0312	0.3854
R_CB11	R_Dorsal_boundary_Zaxis	2.15	0.0332	0.3801
R_CB1	R_CB18	2.15	0.0332	0.3801
R_CB33	R_CB4	2.12	0.0359	0.3748
R_CB28	R_CB24	2.12	0.0359	0.3748
R_CB28	R_CB26	2.11	0.0372	0.3730
R_CB11	R_CB11	2.11	0.0372	0.3730
R_CB1	R_CB26	2.09	0.0384	0.3695
R_CB11	R_Ventral_boundary_Zaxis	2.06	0.0418	0.3642
R_CB1	R_CB5	2.06	0.0418	0.3642
R_CB11	R_CB14	2.04	0.0429	0.3606
R_CB1	R_CB36	2.02	0.0458	0.3571
R_CB22	R_CB21	2.01	0.0463	0.3553
R_CB11	R_CB19	2	0.0472	0.3536
R_CB1	R_CB38	2	0.0472	0.3536
R_CB11	R_CB26	2	0.0472	0.3536
R_CB1	R_CB3	2	0.0472	0.3536
R_CB21	R_CB7	1.99	0.0483	0.3518
R_CB13	R_CB14	-2.39	0.0183	-0.4225
R_CB1	R_CB3	-2.39	0.0183	-0.4225
R_CB11	R_Ventral_boundary_Zaxis	-2.8	0.0058	-0.4950
R_CB12	R_CB39	-2.8	0.0058	-0.4950
R_Lateral_boundary_Xaxis	R_CB7	-3.65	0.0004	-0.6452

R_CB11	R_CB7	-4.98	0.0000	-0.8803
R_CB1	R_CB25	-4.98	0.0000	-0.8803

Note. This table includes ROI-to-ROI FC values in both directions (e.g., R_CB1 to R_CB37 and R_CB37 to R_CB1 if both connections were significant).

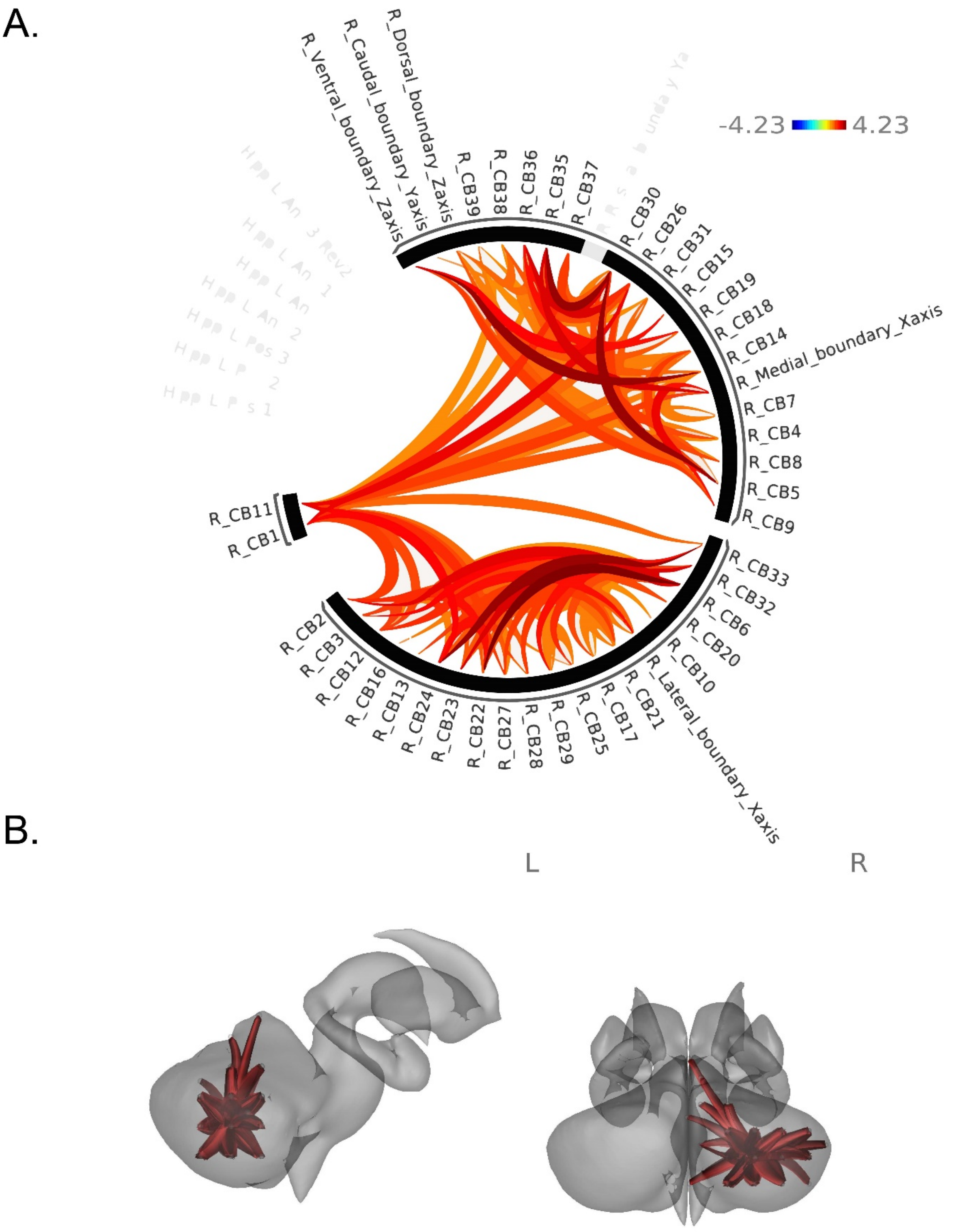

Supplementary Figure 2. Patterns of intracerebellar functional connectivity (FC) between ROIs in males as compared to females, when controlling for age. **A**. ROIs are shown in an FC ring where orange-red displays greater FC in males as compared to females. **B**. ROIs are shown on a

subcortical model where red displays positive intracerebellar FC relationships between ROIs in males as compared to females.

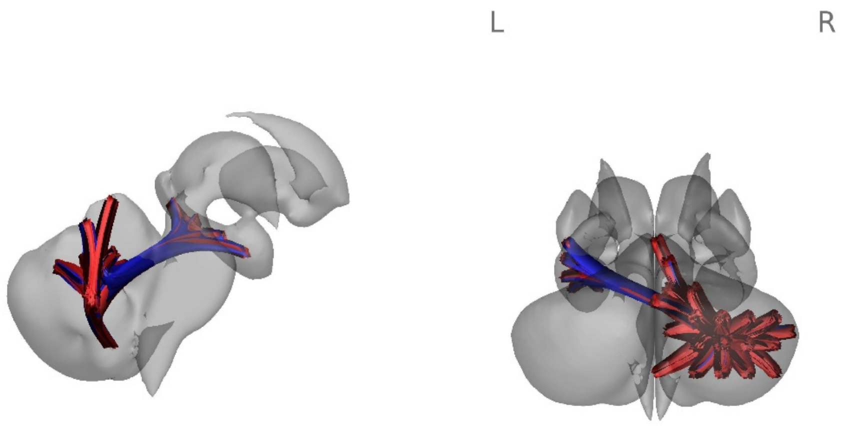

Supplementary Figure 3. Cerebellar and hippocampal regions showing significant within structure correlations in cortical FC with increased progesterone levels, when controlling for age. ROIs are shown on a subcortical model where blue represents negative FC relationships between ROIs with higher progesterone levels, while red displays positive FC relationships between ROIs with higher progesterone levels.

Supplementary Table 7. Behavioral performance relationships with age, education, and sex.

Behavioral Measure	Main Effect of Age (pFDR)	Main Effect of Education Level (pFDR)	Main Effect of Sex (pFDR)	df
Symbol Span	0.005*	0.606	0.918	135
Stroop Task	0.238	0.606	0.763	126
Pegboard Assembly	<0.001*	0.578	0.186	135
Shopping List Memory	0.005*	0.060	0.444	126
Sequence Learning	0.008*	0.682	0.763	104
Letter-Number Sequencing	<0.001*	0.504	0.763	135

Note. * *indicates significant p-value after FDR correction.*

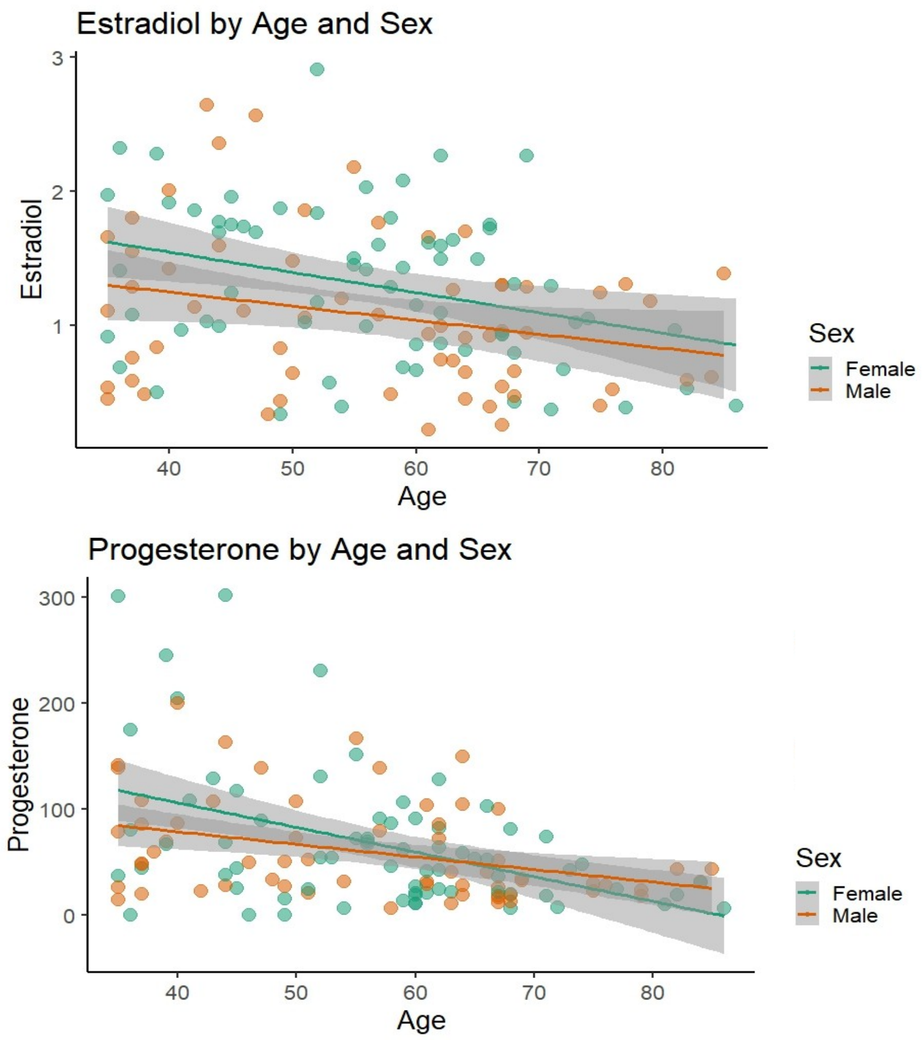

Supplementary Figure 4. Linear regressions between 17-β estradiol (top) and progesterone (bottom) with age by sex. Females are displayed in green and males in orange.
